# Spatial Mapping of Plant *N*-Glycosylation Cellular Heterogeneity Inside Soybean Root Nodules Provided Insights Into Legume-Rhizobia Symbiosis

**DOI:** 10.3389/fpls.2022.869281

**Published:** 2022-05-16

**Authors:** Dušan Veličković, Yen-Chen Liao, Stephanie Thibert, Marija Veličković, Christopher Anderton, Josef Voglmeir, Gary Stacey, Mowei Zhou

**Affiliations:** ^1^Environmental Molecular Sciences Laboratory, Earth and Biological Sciences Directorate, Pacific Northwest National Laboratory, Richland, WA, United States; ^2^Glycomics and Glycan Bioengineering Research Center, College of Food Science and Technology, Nanjing Agricultural University, Nanjing, China; ^3^Divisions of Plant Sciences and Biochemistry, C. S. Bond Life Sciences Center, University of Missouri, Columbia, MO, United States

**Keywords:** MALDI, mass spectrometry imaging, plant, PNGase H, *N*-glycans, proteomics, glycoprotein, soybean root nodule

## Abstract

Although ubiquitously present, information on the function of complex *N*-glycan posttranslational modification in plants is very limited and is often neglected. In this work, we adopted an enzyme-assisted matrix-assisted laser desorption/ionization mass spectrometry imaging strategy to visualize the distribution and identity of *N*-glycans in soybean root nodules at a cellular resolution. We additionally performed proteomics analysis to probe the potential correlation to proteome changes during symbiotic rhizobia-legume interactions. Our ion images reveal that intense *N*-glycosylation occurs in the sclerenchyma layer, and inside the infected cells within the infection zone, while morphological structures such as the cortex, uninfected cells, and cells that form the attachment with the root are fewer *N*-glycosylated. Notably, we observed different *N*-glycan profiles between soybean root nodules infected with wild-type rhizobia and those infected with mutant rhizobia incapable of efficiently fixing atmospheric nitrogen. The majority of complex *N*-glycan structures, particularly those with characteristic Lewis-a epitopes, are more abundant in the mutant nodules. Our proteomic results revealed that these glycans likely originated from proteins that maintain the redox balance crucial for proper nitrogen fixation, but also from enzymes involved in *N*-glycan and phenylpropanoid biosynthesis. These findings indicate the possible involvement of Lewis-a glycans in these critical pathways during legume-rhizobia symbiosis.

## Introduction

Plant glycoproteins have a characteristic and diverse set of *N*-glycans within their structures, which are attached *via* asparagine (N) at specific consensus sequences N-X-(S/T), with the X symbolizing any amino acid except proline (Frank et al., 2021). The pathway for the initial glycan processing and synthesis of core *N*-glycans in the endoplasmic reticulum (ER) is evolutionally conserved in all eukaryotes and is essential for cell viability as part of the protein folding machinery (Nagashima et al., 2018). Maturation of *N*-glycans occurs in the Golgi apparatus, and this stage of glycan synthesis is unique for a specific eukaryotic kingdom. For example, plants synthesize complex *N*-glycans with unique motifs, namely β1,2-xylose (Xyl), core α 1,3-fucose (Fuc), and Lewis a-epitope (β1,3-galactose (Gal) and α1,4-Fuc linked to terminal *N*-acetylglucosamine, GlcNAc) not found in mammalian and other species. In contrast, mammalian epitopes are often sialylated and may contain bisecting β-1,4-GlcNAc structures, core α1,6-linked Fuc residues, or *N*-acetyllactosamines (LacNAc) (Nagashima et al., 2018). The late stage of plant *N*-glycan maturation is also unique to the action of β-*N*-acetylhexosaminidases that remove the two-terminal GlcNAc residues at the non-reducing ends of *N*-glycans, which create paucimannose *N*-glycans. These truncated *N*-glycan structures constitute the majority of glycans present on vacuolar glycoproteins and occur in a smaller amount on extracellular plant glycoproteins (Liebminger et al., 2011).

Contrary to mammals, where complex *N*-glycans are essential in molecular recognition and signaling events, the exact biological function of *N*-glycans in plants– beyond their role in protein folding—has long been neglected, and new findings are still very limited (Strasser, 2016). Recent genetic studies have demonstrated their importance in cell wall biosynthesis, salt stress tolerance, and phytohormone levels and transport (Strasser, 2014, 2016; Kaulfurst-Soboll et al., 2021; Yoo et al., 2021), for example. Moreover, with the extracellular medium in plants being acidic and rich in proteases, extensive *N*-glycosylation stabilizes plant proteins in such harsh conditions (Lefebvre et al., 2012). To better understand plant glycobiology, we need, among other data, information on cell-type or organ-specific occurrence of specific glycan structures. To date, these data are almost entirely missing due to limitations in experimental approaches that can spatially resolve glycan profiles in plant tissues.

The *N*-glycan spatial profiling using matrix-assisted laser desorption/ionization (MALDI) mass spectrometry imaging (MSI) has emerged over the past few years as a powerful approach in clinical research for insight into aberrant *N*-glycan structures in specific cell types and histopathological regions during disease progression (Carter et al., 2020; Conroy et al., 2021; Grzeski et al., 2022; Malaker et al., 2022; Zhang et al., 2022). The method relies on spraying the enzyme peptide *N*-glycanase (PNGase) over formalin-fixed paraffin-embedded (FFPE) tissue sections to *in-situ* release the *N*-linked carbohydrates from their carrier proteins. Released glycans are then spatially probed by MALDI-MSI, which uses a focused laser to ablate and ionize molecules for MS analysis (Drake et al., 2018; Velickovic et al., 2021). In this work, we adapted this approach, by using a unique and novel plant-specific PNGase, to demonstrate its validity, robustness, and depth of coverage for characterizing plant *N*-glycans' spatial patterns and cell-type-specific profile. We used soybean root nodules as the model system to show differences in the *N*-glycan distribution pattern, segregation, and abundance related to nodule capability to perform efficient biological nitrogen fixation (BNF). These specialized root organs have several visually and functionally distinct compartments (i.e., outer cortex, sclerenchyma, inner cortex, and infection zone), where a population of root cells is infected with nitrogen-fixing soil bacteria (Rhizobiaceae) (Livingston et al., 2019). Using the MALDI-MSI (Velickovic et al., 2018) and other MSI modalities (Agtuca et al., 2020; Samarah et al., 2021), we recently spatially resolved critical metabolic and lipidomic pathways in different compartments of soybean root nodules, and also showed different metabolic profiles in infected and uninfected plant cells inside the infection zone (Samarah et al., 2020).

For the symbiotic interaction to occur, legumes first exude chemical signals (e.g., flavonoids and isoflavonoids) that activate rhizobia NodD proteins, which bind to bacterial promoters and induce the expression of several genes leading to biosynthesis and secretion of signaling molecules called Nod factors (Downie, 2010). These signaling molecules are oligomers of usually four or five 1,4-linked *N*-acetyl-glucosamine residues that carry an *N*-linked acyl group (Downie, 2010), which legumes recognize *via* perception motifs that are highly *N*-glycosylated proteins (Lefebvre et al., 2012). To optimize legume infection and to form effective nitrogen-fixing nodules, rhizobia export different surface polysaccharides and proteins, deliver proteins into plant cells and use quorum-sensing regulation of gene expression to coordinate their behavior in ways that enhance and spread their symbiotic capacity (Downie, 2010). As such, *N*-glycans attached to the ectodomains of plasma membrane pattern recognition receptors constitute likely initial contact sites between plant cells and invading pathogens (Haweker et al., 2010). BNF is performed by an oxygen-intolerant nitrogenase enzyme but requires respiration to meet its high energy demand (Rutten and Poole, 2019), and there is a hypothesis that a glycoprotein localized in the inner cortex may control oxygen diffusion into rhizobial-infected cells (James et al., 1991) where leghemoglobin serves as oxygen buffer (Halwani et al., 2021). Despite all these hypotheses on *N*-glycoprotein importance for efficient symbiosis, the *N*-glycome profile of the legume root nodule has never been explored. Herein, we compared the spatial pattern, abundance, and potential protein origin of *N*-linked glycans in soybean root nodules infected with the wild type *Bradyrhizobium japonicum* that enables efficient nitrogen fixation, and soybean root nodules infected with a *Bradyrhizobium japonicum nifH*- mutant incapable of fixing nitrogen (Hahn et al., 1984), to inform on the potential role of protein *N*-glycosylation during BNF and plant-microbe symbiosis.

## Materials and Methods

### Plant Growth and Harvesting

Plant growth was performed at the University of Missouri following procedures described in our previous work (Velickovic et al., 2018). Briefly, rhizobial cells (*Bradyrhizobium japonicum* USDA110 wild-type (WT) and fix-mutant H1 (*nifH*-) were inoculated into HM medium (HEPES, 1.3 g/L; MES, 1.1 g/L; Na_2_HPO_4_, 0.125 g/L; Na_2_SO_4_, 0.25 g/L; NH_4_Cl, 0.32 g/ L; MgSO_4_•7H_2_O, 0.18 g/L; FeCl_3_, 0.004 g/L; CaCl_2_•2H_2_O, 0.013 g/L; yeast extract, 0.25 g/L; D-Ara, 1 g/L; sodium gluconate, 1 g/L; and pH 6.6) (Cole and Elkan, 1973), supplemented with 25 mg/L of tetracycline and 100 mg/L of spectinomycin for wild-type and 100 mg/L of kanamycin and spectinomycin for *nifH-*. The cells were then incubated and maintained for 2 d at 30°C in an orbital shaker (MaxQ400, Thermo Scientific, Waltham, MA, USA) set to 180 rpm. Once cellular growth reached 10^8^ cells/mL, as measured by optical density (OD600 = 0.8), the culture was centrifuged at 800 × g for 10 min, washed three times with DI water, and used for seedling inoculation. Soybean seeds (*Glycine max* cv. Williams 82), sterilized with 20% (v/v) bleach for 10 min and rinsed five times in sterile water, were planted into pots containing a mixture of autoclaved 3/1 vermiculite/perlite, respectively. The plants were grown in a greenhouse at 30°C with a 16 h light/8 h dark cycle, and on day 3 the seedlings (10 in total) were inoculated with 1 ml of *B. japonicum* suspension per seedling on the soil. On day 21 of growth, the roots with attached nodules were freshly harvested, plunged into liquid nitrogen, and stored at −80°C until further use.

### Preparation of Soybean Nodule for MALDI-MSI: Formalin Fixation, Paraffin Embedding, and Sectioning

Three individual wild-type nodules and three individual *nifH*- nodules, each excised from the roots of a separate soybean plant, were soaked in 1mL of 10% formalin for 24 h for fixation. Nodules were then washed in cold running tap water for 30 min and then dehydrated using a series of washing steps: 70% ethanol (EtOH) for 1 h, 80% EtOH for 1 h, 95% EtOH for 1 h, 95% EtOH for 1.5 h, 100% EtOH for 1 h, 100% EtOH for 1.5 h, and xylenes for 1 h, xylenes for 1.5 h. They were then placed in melted paraffin wax (60°C) for 1 h and melted paraffin (60°C) for 1.5 h, before embedding in paraffin blocks. Tissues were then sectioned at 7 μm thickness using a HM 330 microtome (MICROM, Heidelberg, Germany) and mounted on the conductive indium tin oxide (ITO)-coated glass slides (Bruker Daltonik GmbH, Bremen, Germany) for MALDI-MSI analysis. To minimize the batch effects and to ensure that all sections receive the same downstream treatment, sections were mounted in a way that all analyzed samples (all WT bio-replicates and all *nifH-* bio-replicates) were on the same ITO slide, analogous to well-established tissue microarray (TMA) clinical assays (Grzeski et al., 2022).

### PNGaseF and PNGaseH+ Application for *in situ N*-Glycan Releasing

The PNGase F Prime (*N*-Zyme Scientifics, Doylestown, PA, USA) with a specific activity of 500U/mg and acidic PNGase H+ variant Dj with a specific activity of 150 U/mg (Guo et al., 2020) were used in the experiments. Enzyme application was performed according to the recently published protocol (Angel et al., 2018; Drake et al., 2018) with substantial modification in the washing procedure before PNGase H+ application. Briefly, slides were heated at 60°C for 1 h and then dewaxed (xylenes; 2 × 3 min) and rehydrated (100% EtOH, 2 × 1 min; 95% EtOH, 1 min; 70% EtOH, 1 min; H_2_O, 2 × 3 min) for heat-induced antigen retrieval. Slides were then placed in a five-slide mailer with a citraconic anhydride buffer (pH~3) solution, and antigen retrieval was carried out for 30 min using a food steamer (AROMA, San Diego, CA, USA). Before PNGase F application, the citraconic buffer was exchanged with distilled water, and slides were dried in a desiccator. For PNGase H+ application, exchanging with distilled water was omitted, and slides were immediately dried in a desiccator after antigen retrieval to ensure acidic conditions necessary for optimal PNGaseH+ activity. Each enzyme (PNGase F at 100 U/ml and PNGase H+ at 105 U/ml) was sprayed over the slide-mounted tissue samples using a HTX M5 Sprayer (HTXImaging by HTX Technologies LLC, Chapel Hill, NC, USA) at a 25 μl/min flow rate, using 15 passes, and a crisscross pattern, at a 1,200 mm/min spray head velocity, and a 3 mm track spacing. Slides were then placed in a preheated humid incubation chamber and incubated for 2 h at 37°C.

### MALDI Matrix Deposition and MSI Acquisition

Post enzyme releasing steps, α-Cyano-4-hydroxycinnamic acid, CHCA (Sigma-Aldrich, St. Louis, MO, USA) at a concentration of 7 mg/ml (50% ACN with 0.1% TFA) was sprayed over the tissue sections using the TM-Sprayer with the following settings: 100 μl/min, 10 passes, crisscross pattern, a velocity of 1,300 mm/min, 3 mm track spacing. MSI experiments were performed using a 15 Tesla SolariX Fourier-transform ion cyclotron resonance (FTICR) mass spectrometer (Bruker Daltonics, Bremen, Germany) equipped with a dual ESI/MALDI ion source and a Smart-beam II Nd:YAG (355 nm) laser. The instrument was operated in positive ion mode over an m/z range of 1,000–5,000 with an estimated resolving power of 330,000 at m/z 400. The target plate stepping distance was set to 50 μm, which defined the spatial resolution for these measurements (i.e., the laser spot size was smaller than the stage stepping distance). Higher spatial resolution imaging experiments (at 15 μm spatial resolution) were performed using a MALDI source (Spectroglyph, LLC, Kennewick, WA, USA) with a 1 kHz Explorer One Nd:YAG (349 nm) laser (Spectra-Physics, Santa Clara, CA, USA) coupled to a custom ThermoFisher QE-Orbitrap MS (Bremen, Germany) (Zemaitis et al., 2022) in positive ion mode in an m/z range of 600–6,000 with a mass resolution of ~200,000 at m/z 400.

### MALDI Imaging Data Analysis

Imaging data files were imported into the SCiLS lab software, exported without normalization to imzML, and the resulting .imzML and .ibd files were then submitted to METASPACE for data processing and annotation. The NGlycDB (Velickovic et al., 2021) was chosen as the database, with positive polarity and [M+Na]^+^ selected as the ionization adduct. The default METASPACE m/z tolerance of ± 3 ppm was used. ROC (Receiver Operating Characteristic) processing in the SCILS lab was used for determining discriminant (significantly changed in abundance) *N*-glycans (Grzeski et al., 2022) between WT and *nifH*- nodules. ROC is a univariate measure to assess the discrimination quality of m/z values through two groups (in this case all mutant sections as one group and all WT sections as the second group). The ROC is calculated based on the statistical specificity and sensitivity when the intensity of a single feature is used for the discrimination rule. Specifically, we set the AUC threshold values (Area Under the ROC Curve), the parameter used to estimate the discrimination quality of ROC value, as > 0.6 or <0.4 (Kulbe et al., 2020) to determine the upregulated or downregulated *N*-glycan m/z ions, respectively. *N*-glycan signals with AUC values within the 0.4–0.6 range were considered non-significantly changed. The discriminative potential of *N*-glycan structures was classified (Safari et al., 2016) as poor (0.6 ≤ AUC < 0.7), fair (0.7 ≤ AUC < 0.8), good (0.8 ≤ AUC < 0.9), and excellent (AUC ≥ 0.9). The data sets, including their imzML files, are available at https://metaspace2020.eu/project/soybean_nodule_Nglyc_2022.

### Bottom-Up Proteomics

A total of five biological replicates of WT and *nifH*- soybean nodules were prepared for bottom-up proteomics. Using cold mortar and pestle, frozen nodules were homogenized with 470 μl of ice-cold 4:3 methanol:water mixture (v/v). Homogenized nodules were transferred to a tube and further homogenized using a Pellet Pestle homogenizer and kept on ice to stay cold throughout the lysis procedure. 530 μl of ice-cold chloroform was added to each sample. All samples were vortex mixed for 1 min, following incubation on ice for 5 min, then vortexed for 1 min again. Samples were centrifuged at 10,000 g at 4°C for 10 min. The upper aqueous phase and lower organic phase were removed. The interphase, which contains proteins, was washed with 1 mL of ice-cold methanol, vortex mixed for 1 min, and centrifuged at 10,000 g at 4°C for 10 min. The supernatant was discarded, and pellets were air-dried for 5 min. Proteins were resuspended in 200 μl of lysis buffer (8M urea buffer, 50 mM ammonium bicarbonate buffer, pH 8). 1:20 (v/v) of 100 mM dithiothreitol (DTT) were added with final concentration 5 mM at 37°C 1,200 rpm for 30 min. 1:20 (v/v) of 200 mM iodoacetamide was added with final concentration 10 mM and react under foil-covered at 50°C 1,200 rpm for 45 min. The solution was diluted to 4M urea by 50 mM ammonium bicarbonate (ABC). Lys C was added with 1:100 (LysC: protein, w/w) and react at 37°C for 4 h. The solution was further diluted to 1M urea by 50 mM ABC. Trypsin was added at a concentration of 1:50 [trypsin: protein (w/w)] and then left to react at 37°C overnight. The peptide was cleaned by C_18_ solid-phase extraction (SPE) and submitted to LC-MS/MS. The analytical column (75 μm i.d., 30 cm length) was packed with 1.7 μm^2^ C18 beads (Waters BEH) and 200 nl per minute flow. Samples were infused with 0.1% formic acid at 3 μL/min for 5 mins while loading the SPE. We use NanoAcquity UPLC with dual pump trapping mode with 0.1% formic acid in H_2_O (buffer A) and 0.1% formic acid in acetonitrile (buffer B). The LC gradient was programmed as 120 min gradient from 6 to 20% buffer B for 90 min and increased to 60% in 10 min. The column was then washed with 90% buffer B for 5 min and back to 50% for 10 min. The sample was analyzed by Thermo Fisher Orbitrap Eclipse tribrid mass spectrometer. The electrospray was set as 2,400 V with 300°C in an ion transfer tube. MS1 was set with an m/z range of 400 to 2,000, 50 ms maximum injection time, 800,000 AGC target, 30% RF lens, and 120 K resolution at m/z 200. MS2 was set in HCD activation mode with 1.4 as an isolation window and 20, 30, and 40% collision energy: 86 ms maximum injection time, 1,250,000 AGC target, and 50 K resolution at m/z 200.

### Bottom-Up Proteomics Data Analysis

The raw data files for the five WT and *nifH*- biological replicate LC-MS/MS runs were processed using FragPipe v17.1. The protein sequence database file included soybean (UniprotKB release 2021_05, *Glycine max* Proteome UP000008827) and bacterial (UniprotKB release 2021_05, *Bradyrhizobium diazoefficiens* Proteome UP000002526) protein sequences, and reverse decoy sequences and common contaminants that were added within the FragPipe pipeline, as described previously (Kong et al., 2017; Teo et al., 2021). In MSFragger, the precursor mass tolerance was set to −10 to 10 ppm, fragment mass tolerance was set to 20 ppm, mass calibration and parameter optimization were selected, and the isotope error was set to 0/1/2. “Stricttrypsin” was selected as the enzymatic cleavage, with peptide length from 7 to 50 amino acids and peptide mass ranging from 400 to 8,000 Da. Under modifications, the variable modifications enabled were methionine oxidation and *N*-terminal acetylation, with the maximum number of variable modifications on a peptide set to 3 and maximum combinations set to 5,000. Cysteine carbamidomethylation was selected as a fixed modification. Mass offsets included “0” to account for no offset, and all masses corresponding to mass shifts associated with 52 common plant *N*-glycans, obtained from Protein Metrics Inc. Byonic software v15.2.1 (Bern et al., 2012) and the *N*-glycans observed in MALDI data (Supplementary Table 1). The labile modification search mode was set to N glycan with the diagnostic ion minimum intensity set to 0.1. The top 300 most intense peaks and a minimum of 15 fragment peaks were used for spectral processing. PTM-Shepherd was run with 0.01 Da precursor tolerance, 0.002 Da peak picking width, localization restricted to N, and all other parameters set to the defaults for glyco search. IonQuant was run using MS1 quantification defaults. Philosopher v4.1 was used for data analysis (Leprevost et al., 2020).

Within the Fragpipe combined modified peptide output file, qualitative analysis of the proteins found to contain *N*-glycans was performed, with the proteins found to contain Lewis-a glycans selected for further analysis. Glycopeptides of interest were manually confirmed using the output from Bionic. Briefly, the trypsin specific cleavage residues were specified, maximum common (protein N term acetylation, C and M oxidation, and C dioxidation, trioxidation, and carbamidomethylation) and rare modifications (52 common plant N glycans and the N glycans observed in MALDI data) were each set to 1, and common contaminants and decoys were added. From the combined protein quantitation file, the soybean and bacterial protein data were separated prior to uploading into Perseus v1.6.15 data analysis software (Tyanova et al., 2016). Within Perseus, the uploaded data were log2 transformed, filtered to only include rows containing three or more data values in at least one group, and missing values were replaced separately for each column with a width of 0.3 and downshift of 1.8. These values were then normalized based on the WT and NIF median values and scaled to account for global protein abundance variation between the WT and NIF samples in Excel. For the soybean protein data, a Student paired, two-tailed, *t*-test was performed and the negative natural logarithm of the resultant p-values was calculated. These values, along with the difference between the average NIF and average WT values, were used to generate a volcano plot within Excel, with the proteins found to contain Lewis-a glycans highlighted.

### Pathway and Network Analysis

Protein association networks for the identified proteins were analyzed by STRING database (version 11.5) (Szklarczyk et al., 2019) for high-confidence (score > 0.7) and a medium-confidence (0.4 < score < 0.7) protein-protein interactions. Functional enrichment analysis was performed by ClueGO plugin (version 2.5.8) (Bindea et al., 2009) in Cytoscape (version 3.8.2) (Shannon et al., 2003) against the gene ontology (GO) (Ashburner et al., 2000) and Kyoto Encyclopedia of Genes and Genomes (KEGG) database (Kanehisa and Goto, 2000; Kanehisa et al., 2021) with soybean (*Glycine max*) and bacteria (*Bradyrhizobium diazoefficiens*) protein database.

## Results

### Enzyme Selection for Soybean Nodule *N*-Glycan Spatial Profiling

To obtain maximal coverage of *N*-glycans from the soybean root nodules, we tested and compared commercially available recombinant PNGaseF PRIME that has been widely used in *N*-glycan MALDI-MSI of mammalian tissues (Powers et al., 2014; Carter et al., 2020; Blaschke et al., 2021; Mcdowell et al., 2021) and acidic PNGase H+ that has previously shown high specificity in bulk plant *N*-glycan profiling (Du et al., 2015). For assessing their efficiency, we concurrently sprayed these enzymes over the cross-section of wild type and *nifH*- FFPE soybean root nodule, analyzed released glycans by MALDI-MSI, and automatedly annotated *N*-glycans using the NGlycDB (Velickovic et al., 2021) in METASPACE. METASPACE is an open cloud software platform that performs molecular annotations for MSI data—based not only on the accurate mass information but also on a comprehensive bioinformatics framework that considers the relative intensities and spatial colocalization of isotopic peaks, as well as quantifies spatial information with a measure of spatial chaos followed by the estimation of the False Discovery Rate (FDR) (Palmer et al., 2017). Recently, we introduced NGlycDB which contains naturally occurring *N*-linked glycans, including all reported plant glycans, for researchers worldwide to annotate their spatial *N*-glycomics using METASPACE (Velickovic et al., 2021).

Our results indicate that PNGase H+ outperformed PNGase F by every metric, as it released more types of glycans with core fucoses and more types of glycans with xylose and Lewis-a structures that are unique epitopes of plant glycoproteins (Table 1). In addition, we also found that FFPE treatment was critical for successful *N*-glycan plant imaging. Specifically, we tested the efficiency of our workflow on fresh frozen soybean nodules, but the results were abysmal, where we were unable to annotate any *N*-glycans from the soybean nodule tissue. We posit this is because many glycoproteins were washed away during tissue treatment—as they were not crosslinked as in FFPE samples—or glycans were not adequately exposed to the PNGase enzyme after the dehydration step (Powers et al., 2014). These findings are consistent with what was observed in MALDI-MSI analysis of mammalian tissue (Powers et al., 2014).

**Table 1:** The number of annotated *N*-glycan species from a single cross-section of *nifH-* and WT soybean root nodule after PNGaseF and PNGaseH+ treatment, provided by METASPACE using the NGlycDB and FTICR MS analysis.

**# Annotations (NGlycDB)**	**PNGaseF**		**PNGaseH**	
	***nifH-***	**WT**	***nifH-***	**WT**
Total	28	17	33	26
Not possible^a^/out of the tissue^b^	2	3	1	4
Total real	26	14	32	22
With core fucose^c^	8	4	17	10
With xylose	16	9	22	16
With Lewis a structure	4	0	7	4
Oligomannosidic	5	5	7	5

*Some of the compositions are falsely annotated as indicated by*

a *N-glycan structure that is annotated as sialylated glycans (characteristic of mammalians glycans) and*

b *features that are out of the tissue*.

c *Only considered if all isomers from NGlycDB contain core fucose*.

### Soybean Root Nodules Without Nitrogen-Fixing Capability Show an Increased Level of *N*-glycosylation

**Figure 1:**
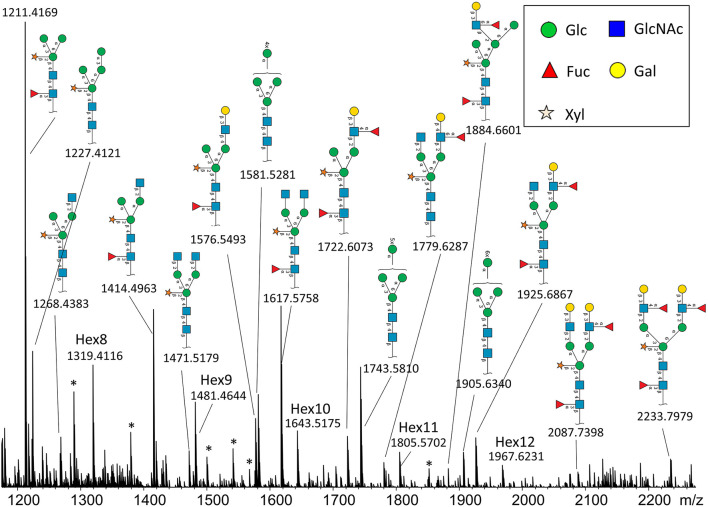
Average MALDI imaging spectrum and Symbol Nomenclature for Glycans (SNGF) annotation of the most intense *N*-glycans from nifH- soybean root nodule treated with PNGaseH+. Note that some peaks are annotated as hexose oligomers (Hex8-Hex12), and they probably originate from starch or cellulose. There are also some unidentified peaks (labeled with *).

**Figure 2:**
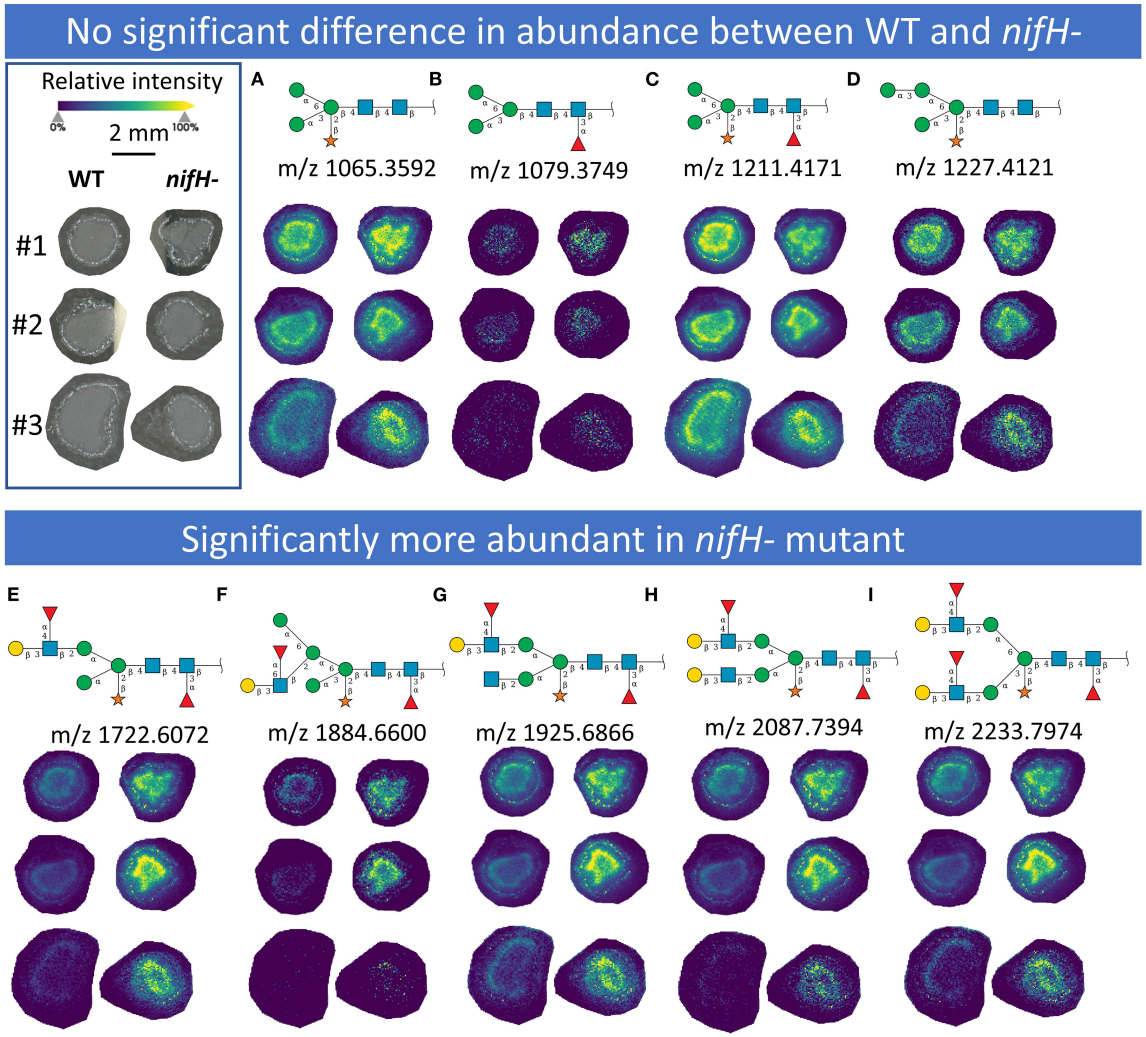
MALDI MS images of exemplary *N*-glycans that (upper panel) show no significant differences in abundance between WT and *nifH-*mutant **(A–D)** and those (lower panel) that are significantly more abundant in *nifH*- mutant **(E–I)**. For each *N*-glycan ion images, color scale bar was created separately. Representative Symbol Nomenclature for Glycans (SNGF) and distribution patterns of three bioreplicates (#1, #2, and #3) at 50 μm spatial resolution are shown. Glycan compositions of representative SNGF: **(A)** Hex:3 HexNAc:2 Pent:1, **(B)** Hex:3 HexNAc:2 dHex:1, **(C)** Hex:3 HexNAc:2 dHex:1 Pent:1, **(D)** Hex:4 HexNAc:2 Pent:1, **(E)** Hex:4 HexNAc:3 dHex:2 Pent:1, **(F)** Hex:5 HexNAc:3 dHex:2 Pent:1, **(G)** Hex:4 HexNAc:4 dHex:2 Pent:1, **(H)** Hex:5 HexNAc:4 dHex:2 Pent:1, **(I)** Hex:5 HexNAc:4 dHex:3 Pent:1. Ion images of other *N*-glycans are provided in Supplementary Figures 1, 2.

**Figure 3:**
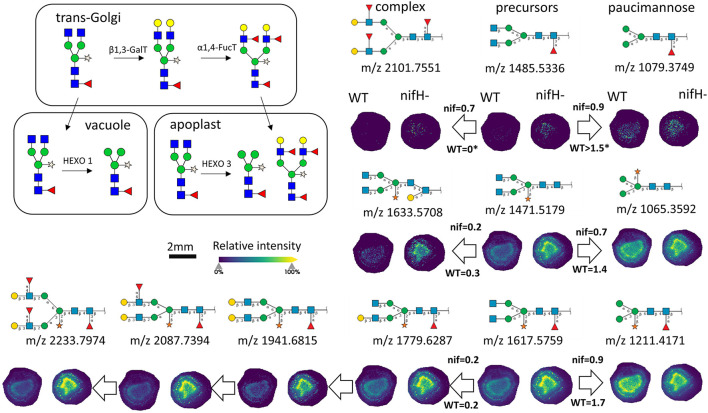
Late stages of *N*-glycan maturation: Lewis-a epitope decoration in trans-Golgi and truncation of *N*-glycans in vacuole and apoplast to paucimannose structures (adapted from Bosch et al., 2013), and ion images of corresponding *N*-glycans involved in the late stages of maturation are illustrated. On the arrows are displayed intensity ratio between the corresponding *N*-glycan signal pairs (ea. paucimannose/precursor and complex/precursor) in the WT and *nifH-* nodules, showing that truncation reaction is more intense in WT nodules. For each *N-*glycan ion image, a color scale bar was created separately.

In total, we were able to image and confidently annotate 34 different *N*-glycan compositions. The most abundant ones are depicted in the overall MALDI mass spectrum in Figure 1. Notably, we also observed hexose oligomers that are most probably starch products. The imaging modality of our approach revealed that all glycans followed a typical spatial pattern: the highest abundance of *N*-glycans was localized in areas that corresponded to the infection zone, whereas some highly abundant *N*-glycan structures were observed in the cortex as well. We first examined the difference in total abundance of *N*-glycans in WT and *nifH-* mutant nodules by performing the receiver operating characteristic (ROC) analysis that is used in tissue microarrays (TMA) for quantifying *N*-glycan differences between clinical samples (Mittal et al., 2021; Grzeski et al., 2022). Herein, we consider all WT nodule sections as one group and all *nifH*- nodule sections as another group. The ROC analysis (Supplementary Table 2) revealed that the majority of *N*-glycans (24) showed an overall higher abundance in the *nifH-* mutant nodules, only a few *N*-glycans (10) showed non-significant changes between WT and *nifH*- nodules, while no *N*-glycan was found significantly more abundant in WT (Supplementary Table 2). Spatial distributions of selected *N*-glycans are illustrated in Figure 2 (complete list is provided in Supplementary Figures 1, 2), where ion map intensities over 3 bio-replicates of *nifH*- and WT nodules clearly show two different abundance relationships of *N*-glycan in two types of nodules. Notably, all glycans with Lewis a-epitope (Figures 2E–I) showed significantly higher abundance in the *nifH-* mutant nodule tissue. In contrast, the level of truncated *N*-glycans (Figures 2A–C) was conserved between the two types of nodules. Insight into the relative abundance between Lewis-a type, truncated *N*-glycans, and their common precursors revealed that the truncation yield was higher in the WT compared to *nifH-* mutant nodules, as observed in Figure 3. In contrast, it seems that the conversion rate to complex *N*-glycans is similar in WT and mutant nodules. Lastly, it seems that oligomannosidic glycans (data shown in Supplementary Figure 2) are all significantly upregulated in the mutant nodules. For simplicity, each *N*-glycan composition is represented with only one tentative *N*-glycan structure annotated by the naturally occurring *N*-glycan database (NGlycDB) in METASPACE, but we don't exclude the possibility that other mass isomers are present as well. All tentative structures for each composition can be browsed and visualized in our METASPACE project (https://metaspace2020.eu/annotations?db_id=353&ds=2021-08-27_01h19m53s) as illustrated for the example of Hex4 HexNAc2 Pent1 in Supplementary Figure 3.

### Insight into the Fine Spatial Distribution of *N*-glycans in Soybean Root Nodules

**Figure 4:**
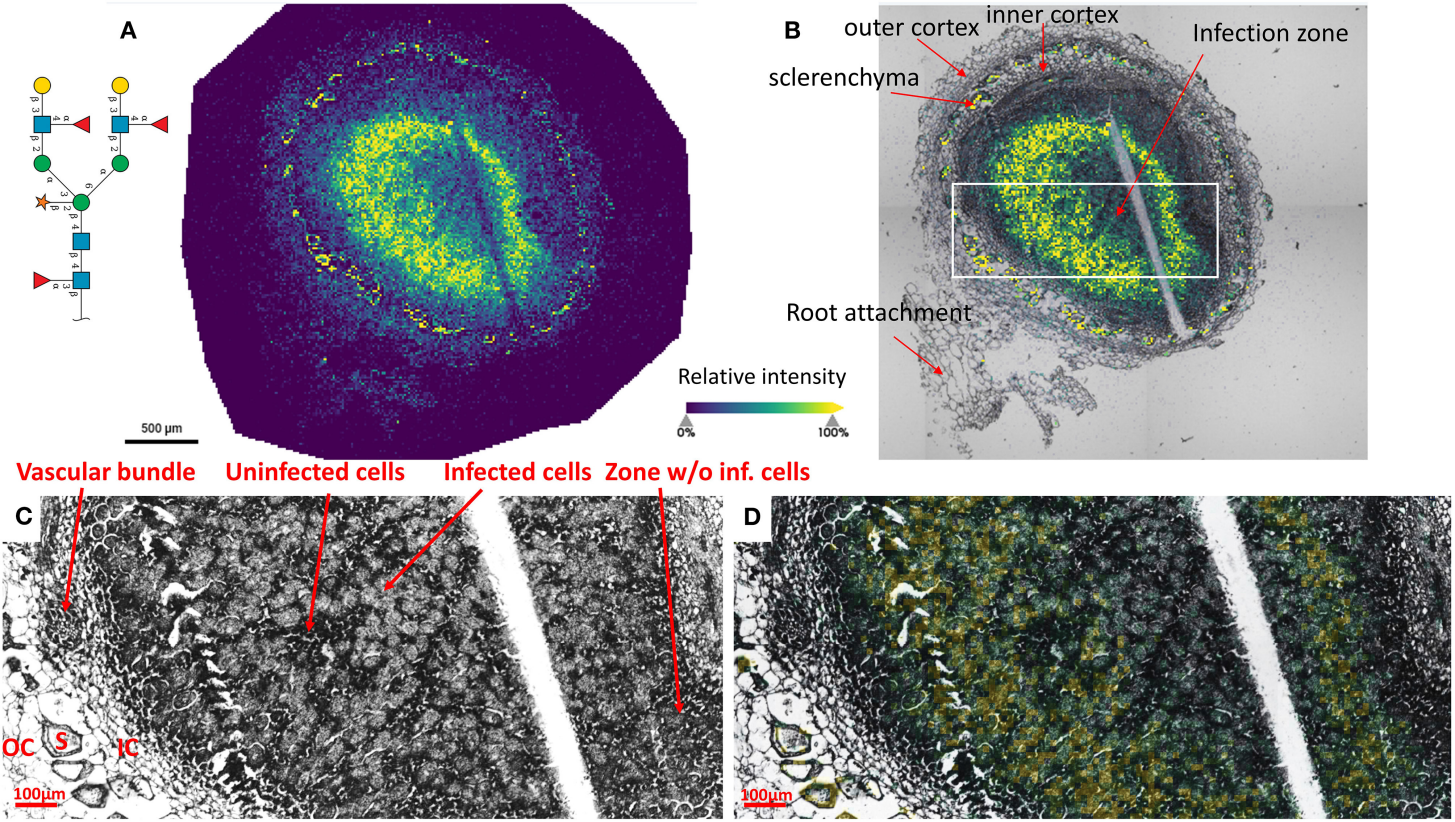
**(A)** Spatial pattern of Hex:5 HexNAc:4 dHex:3 Pent:1 glycan in *nifH-* nodule at 15 μm spatial resolution. **(B)** Morphologically and functionally different cell types and zones inside soybean root nodules are annotated. A white rectangle denotes an area that is enlarged on **(C,D)** for better visualization of fine morphological details. **(C,D)** Zoomed area inside the infection zone with annotated infected and uninfected cells indicate a higher abundance of *N*-glycans in the infected cells (bigger and lighter cells) compared to non-infected cells (smaller and darker). Vascular bundles, inner cortex (IC), outer cortex (OC), and sclerenchyma (S) cells are also annotated. Glycan signal was seen primarily in the infected cells. Some glycan signal was also seen in the sclerenchyma. The scale bar at A and B is 500 μm and at C and D is 100 μm. Note that a thin crack along the infection zone is a sectioning artifact and is an indication that there is no delocalization of the released molecules during the incubation step as no *N*-glycan signal has been observed in this zone. Interactive display of *N*-glycans composition, structure, spatial localization, and colocalization with specific cell types can be further browsed, visualized, and explored using the METASPACE link: https://metaspace2020.eu/annotations?db_id=353&ds=2021-08-07_00h25m23s.

**Figure 5:**
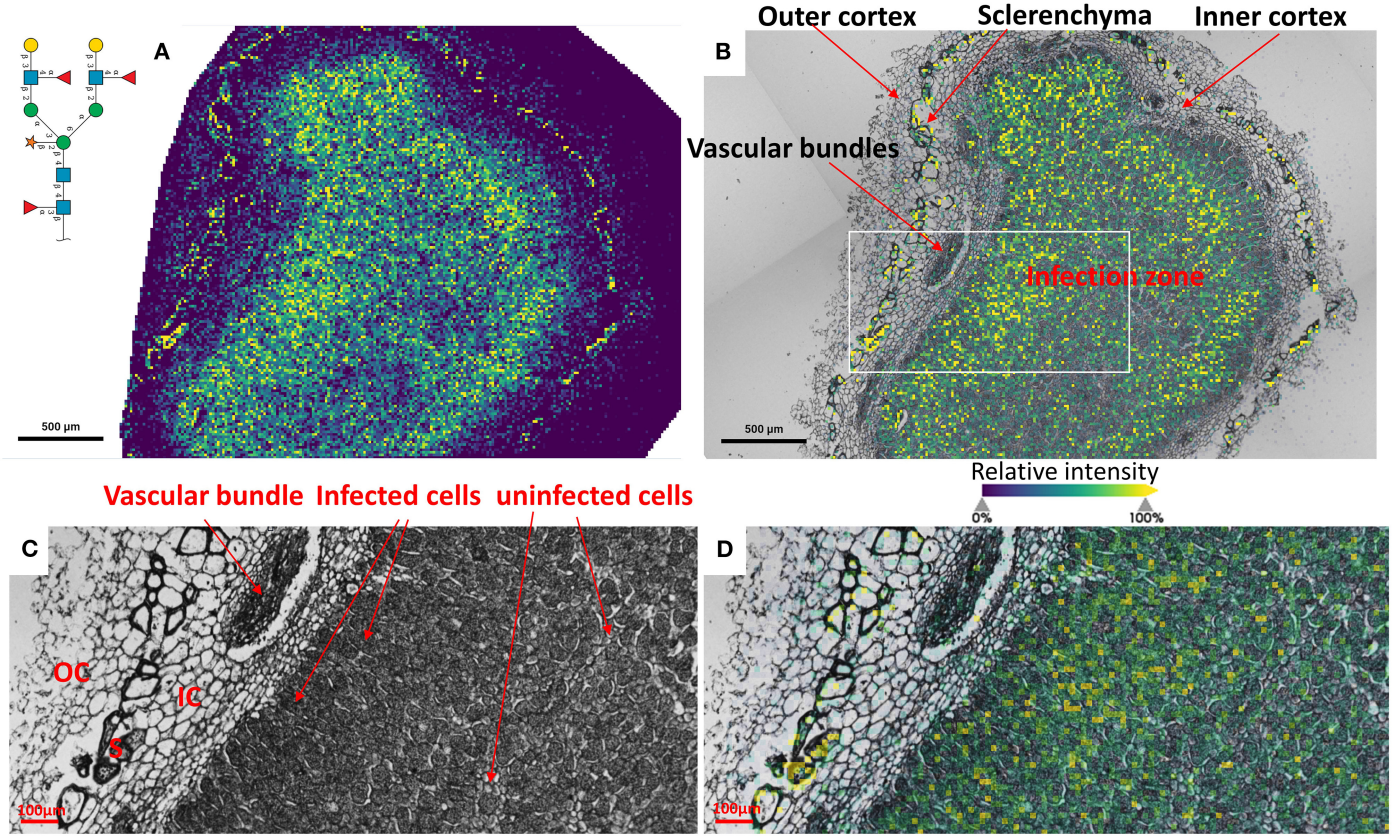
**(A)** Spatial pattern of Hex:5 HexNAc:4 dHex:3 Pent:1 glycan in WT nodule at 15 μm spatial resolution. **(B)** Morphologically and functionally different cell types and layers inside soybean root nodules are annotated. A white rectangle denotes an area that is enlarged on **(C,D)** for fine visualization of distinct cell types. **(C,D)** Zoomed areas inside the infection zone with annotated infected (dark, large cells) and uninfected cells (small light cells) show a higher abundance of *N*-glycans in the dense population of infected cells not interrupted by uninfected cells. Outer cortex (OC), inner cortex (IC), and sclerenchyma cells (S) are also annotated. The scale bar at **(A,B)** is 500 μm and at **(C,D)** is 100 μm. Interactive display of *N*-glycans composition, structure, spatial localization and colocalization with specific cell types can be further browsed, visualized, and explored using the METASPACE link: https://metaspace2020.eu/annotations?db_id=353&ds=2021-08-07_02h15m31s.

To reveal the distributions of *N*-glycans at the cellular level, and to better understand localized glycosylation in specific areas of the infection zone, we analyzed another *nifH-* mutant and WT soybean root nodule at 15 μm spatial resolution. *N*-glycans showed the same distribution pattern as the data collected at 50 μm resolution (Figure 2), but now we could more precisely assign *N*-glycans to specific cell types inside cellularly distinct morphological regions of the soybean root nodule. In *nifH-*, at this finer resolution (Figures 4A,B), glycans were seen to be predominantly localized at the sclerenchyma, the layer of thick-walled cells (typically ~40–50 μm) that separates the outer from the inner cortex (Selker and Newcomb, 1985). Cortex, vascular bundles, and plant cells that form an attachment to root exhibit lower glycosylation compared to sclerenchyma. Inside the infection zone, there was also heterogeneity in glycosylation. In this zone, we can clearly differentiate infected from non-infected cells as infected cells are more voluminous, and lighter in color (Figure 4C), while non-infected cells are smaller and darker (Figure 4C). We observed a ring-like glycosylation spatial pattern inside the infection zone itself, with areas at the periphery and very center showing a decreased level of glycosylation. Overlaying brightfield (Figure 4C) with ion image (Figure 4D) reveals that the more abundant glycosylation co-localizes with infected cells as indicated by higher signal intensity (i.e., yellow-colored) pixels that match infected cells localization, while smaller darker cells show lower *N*-glycan signal intensity (i.e., pixel coloration goes toward greenish). In the center of the nodule, where aggregation of uninfected cells occurs regularly (Selker and Newcomb, 1985) we also saw a decrease in glycosylation. However, in the very center of the infection zone, we also find a group of infected cells with a lower abundance of glycosylation, suggesting that there is glycosylation heterogeneity even among individual infected cells depending on their location in the infection zone. The periphery of the infection zone in the analyzed *nifH-* nodule section almost completely lacks infected cells and glycosylation is decreased in these micro-areas as well. Unexpectedly, we were not able to observe any higher level of paucimannose glycans in the uninfected cells, although almost the entire volume of these cells is occupied by large vacuole (Selker and Newcomb, 1985) where truncation of glycans can occur (Bosch et al., 2013). Spatial analysis of *N*-glycans in the WT nodules leads to similar findings although the infection zone had very distinct structural organization and cell morphology from the mutant. In WT nodules, the coloration of infected cells is different compared to *nifH-* as infected cells of WT nodules have leghemoglobin red pigmentation (Agtuca et al., 2020) so these cells appear as large dark cells under the microscope (Figure 5). Also, the distribution of infected cells seems to be more uniform in WT, with only a few zones intercepted by uninfected cells. Here, however, there is no striking depletion of *N*-glycan abundance in the center of the nodule as it was observed in the mutant, although there is still some decrease compared to the infection zone periphery. The highest level of glycans was again observed in the sclerenchyma layer and in the areas of infected cells uninterrupted by uninfected cells, that is, in the analyzed section, at the outer layer of the infection zone. As in the case of *nifH-* nodules, there was a relatively low level of *N*-glycans in the cortex and vascular bundles of WT nodules (Figure 5).

### Proteomics Analysis Revealed the Increased Abundance of Several Plant Glycoproteins in the Mutant Nodule

**Figure 6:**
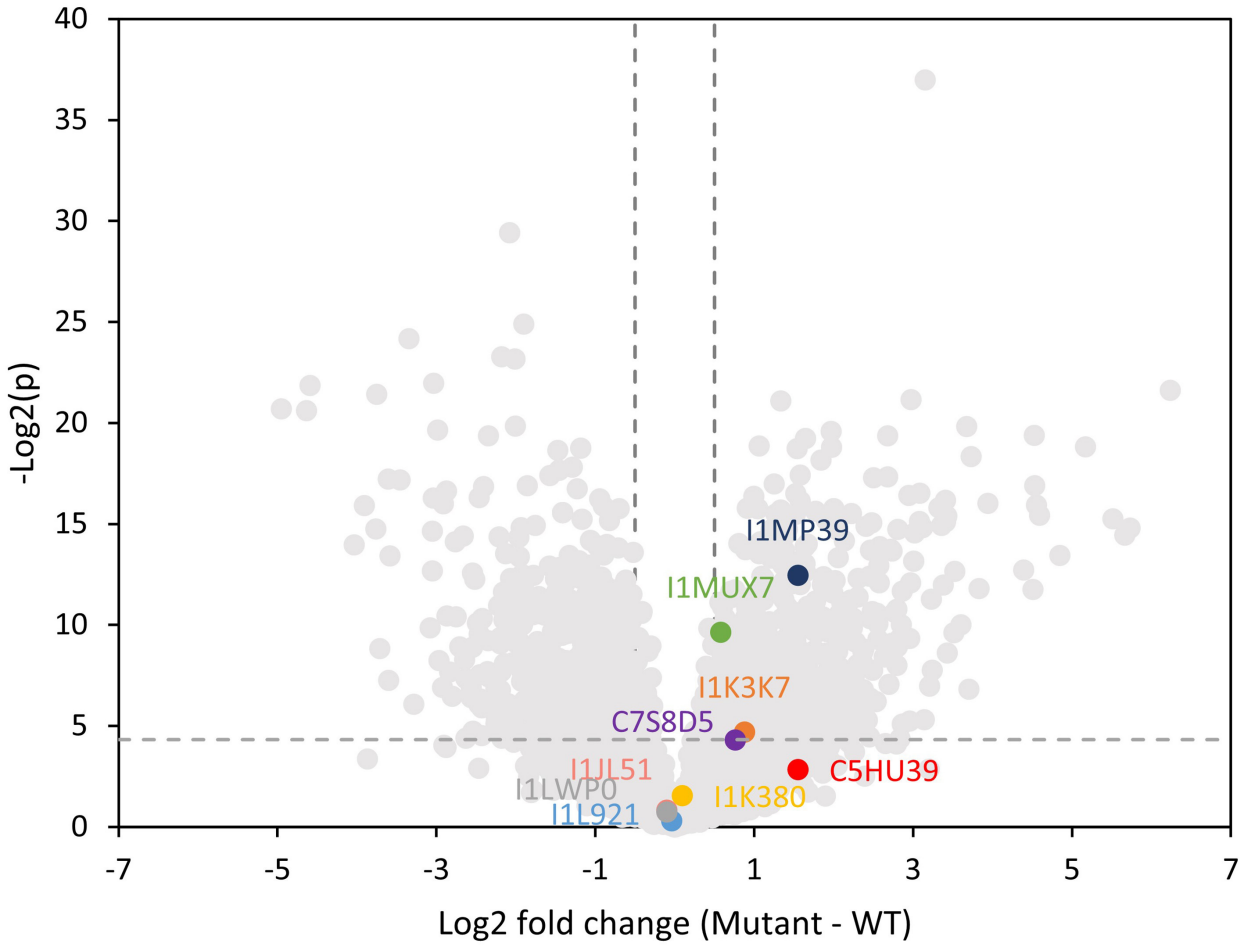
Volcano plot showing log2 fold change in the plant proteome abundance between wild type and mutant soybean nodule. Data points that are above the dashed line [y-value greater than –Log(0.05)] are considered statistically significantly different. Vertical dashed lines mark the 1.4 fold change cutoff (log2 fold change = 0.5). Lewis-a containing glycoproteins are highlighted indicating that three of them are significantly higher abundance in the mutant nodule.

We performed a global proteomics analysis on the whole nodule samples to capture proteome changes. Overall, 6,516 proteins were identified, including 3,653 from plants and 2,864 from the bacterium. We separately examined the soybean and Bradyrhizobium proteomes and selected proteins with log2-fold changes of *nifH-*mutant/wt ratio >0.5 or < -0.5 and *p* < 0.05 into KEGG and biological process gene ontology pathway databases (Supplementary Figure 4; Supplementary Table 3). As a confirmation, the nitrogen fixation process was significantly upregulated in the WT rhizibia proteome, consistent with the fact that the *nifH-* mutant can't fix nitrogen. Generally, we observed an increased abundance of proteins in metabolic pathways in the WT plant and rhizobium. In contrast, oxidative stress-related pathways appeared to be upregulated in the plant infected by *nifH-* mutant, which may be correlated with soybean nodule senescence (Evans et al., 1999). For rhizobial proteins, the ribosome translational machinery, and notably, quorum sensing was upregulated in the mutant. Quorum sensing is involved in the restriction of nodulation (Jitacksorn and Sadowsky, 2008), consistent with the relatively smaller size of the mutant nodules. Within the proteomics data, 89 *N*-glycopeptides from 42 different soybean glycoproteins were also identified (Supplementary Table 4). Since we did not perform glycopeptide enrichment, these identified glycopeptides represent highly abundant glycoproteins, which presumably contributed to the majority of the released *N*-glycans detected with the MALDI-MSI method. Note that 16 unique *N*-glycans detected in glycopeptide data were also observed by MALDI-MSI. Several identified glycoproteins (I1MP39, I1MUX7, and I1K37) were significantly more abundant in the *nifH-* mutant (Figure 6), implying that higher glycan levels observed in the MALDI-MSI data could be a consequence of the increased abundance of certain glycoproteins.

Herein, we specifically focused on glycoproteins that bear one or more Lewis-a epitope and paucimannose glycans, as these classes of glycans showed significant changes in the MALDI-MSI data. Glycopeptide data showed three distinct Lewis-a epitopes: Hex:5 HexNAc:4 dHex:3 Pent:1 (note that the detailed spatial distribution of this glycan is depicted at Figure 4), Hex:4 HexNAc:4 dHex:2 Pent:1 (m/z 1925.687), Hex:4 HexNAc:3 dHex:2 Pent:1 (m/z 1722.607), where Hex:5 HexNAc:4 dHex:3 Pent:1 (m/z 2233.797) were the most frequently detected species, present on 12 peptides from 9 proteins (MS/MS spectra of representative glycopeptides are annotated in Supplementary Figures 5A–I). These nine proteins are identified as phytocyanin domain-containing protein (I1LWP0), amidohydro-rel domain-containing protein (I1L921), dirigent protein (I1JL51), peroxidase (I1MP39), germin-like protein (C7S8D5), an uncharacterized copper ion binding protein with oxidoreductase activity (I1MUX7), an uncharacterized enzyme with mannosyl-oligosaccharide glucosidase activity (I1K3K7), and two uncharacterized proteins with unknown molecular function (I1K380, and C5HU39). Note that phytocyanin (I1LWP0) also contains two detected glycopeptides with Lewis-a group Hex:4 HexNAc:4 dHex:2 Pent:1, while germin (C7S8D5) contains peptide with Hex:4 HexNAc:3 dHex:2 Pent:1 Lewis-a group. All three Lewis-a *N*-glycans co-localize strongly (see Figure 2) and show the exact same trend (in distribution and relative abundance) in our MALDI-MSI data.

**Figure 7:**
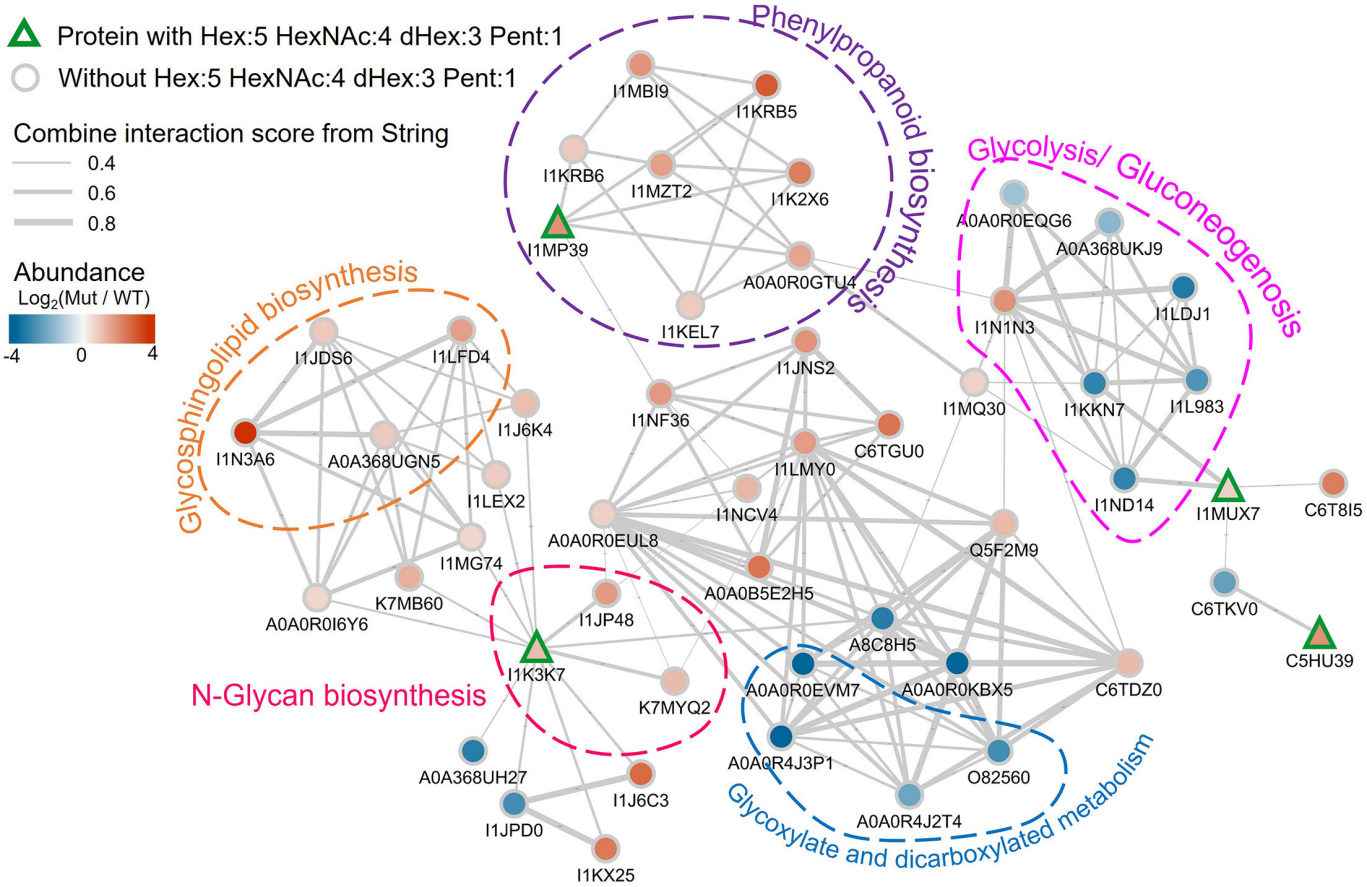
Protein-protein interaction network plot involving the detected Lewis-a containing soybean glycoproteins in the proteomics data, labeled by their UniProt accessions. The proteins with Hex:5 HexNAc:4 dHex:3 Pent:1 are annotated with rectangles with green outlines. Other proteins are shown as gray outlined circles. The fill color of the symbols represents the log2 abundance change in the mutant vs. wildtype, with the color scale in the top left. The gray lines indicate known interactions between the proteins in the String database. The thickness of the line is scaled with their interaction scores (range is from 0.4 to 0.8 as shown by the legend in the top left). Proteins in specific pathways are highlighted with dashed shapes.

Three of these nine proteins (I1MP39, I1K37, and I1MUX7), contain peptides with Hex:5 HexNAc:4 dHex:3 Pent:1 (m/z 2233.797) modification, were significantly higher abundance in the *nifH-* mutant root nodules (Figure 6). We annotated them and their interacting proteins, which had significantly higher expression in *nifH-* mutant than the WT [Log2(M-WT) > 0.5 or < 0.5, *p* < 0.01], with pathways in the KEGG pathway database (Figure 7). Peroxidase (I1MP39) and its interacting proteins are involved in phenylpropanoid biosynthesis (purple area) and have higher expression in the mutant. I1K3K7 participates in the *N*-glycan biosynthesis (red area) with dolichyl-diphosphooligosaccharide-protein glycosyltransferase subunit 2(K7MYQ2 and I1JP48). I1K3K7's interactors—I1JDS6, I1LFD4, I1N3A6, and A0A368UGN participate in glycosphingolipid biosynthesis (orange area). 1MUX7‘s interactor, pyrophosphate-fructose-6-phosphate 1-phosphotransferase (I1ND14 and I1KKN7), is involved in glycolysis/gluconeogenesis (pink area). These proteins and their interactors were suppressed in the mutant nodules while I1MUX7 identified with Hex:5 HexNAc:4 dHex:3 Pent:1 showed higher expression. Notably, neither of these proteins has been documented to bear Lewis-a glycan structure in the UniProt database. The opposite abundance profiles between the two identified glycoproteins and other proteins in the pathway may indicate novel glycosylation-related mechanisms that could be further investigated.

## Discussion

In this work, we adopted the enzyme-assisted MALDI-MS imaging strategy for visualizing the distribution of *N*-glycans in plant tissues at a cellular level using soybean root nodule as a model system. This is an appropriate model sample for exploring cell-cell variability of the *N*-glycosylation posttranslational modifications due to the heterogeneous population of plant cells composing this symbiotic organ. Moreover, although glycans are significant mediators of the nodulation process and plant-microbe interactions, there are no studies on whether protein *N*-glycosylation is affected by nitrogen fixation. Therefore, we compared *N*-glycome spatial and abundance profiles of wild type and *nifH*- mutant soybean nodules, because the *nifH-* mutant still infects the root and forms the nodules, but cannot fix the nitrogen. Such a comparison gave us a unique opportunity to address this open question regarding the impact of glycosylation levels on nitrogen fixation.

The essence of the approach is the *in-situ* releasing of the plant *N*-glycans from their protein carriers so that these carbohydrate structures remain in their original location in the plant tissue. The most widely applied enzyme for *N*-glycans releasing is recombinant PNGase F (from *Flavobacterium meningosepticum*) which shows high specificity toward all types of *N*-glycans, except structures bearing core-linked α 1,3-Fucose (Fan and Lee, 1997) which are often seen in plants and insects. Therefore, plant *N*-glycans are generally released using PNGase A, which is efficient only on small glycopeptides, so the preliminary treatment of the samples with proteases is required. This additional step would introduce complexity in the imaging workflow due to a significantly prolongated pipeline, increased risk of glycan delocalization, and complicated resulting mass spectrum. Recently, Wang et al. (2014) discovered a novel extremely acidic bacterial *N*-glycanase, PNGase H+ from *Terriglobus roseus*, which has the combined advantages of PNGases F and A, because it can utilize glycoproteins with core α-1,3-Fucose as the substrate. Bacterial PNGase H+ variants are structurally related to PNGase A variants in plants such as almonds, rice, and soybean (Supplementary Figure 6), and catalyze the hydrolysis of a β-aspartylglucosaminyl bond between the core-chitobiose region of the *N*-glycan and the asparagine (Asn) residue. However, bacterial PNGase H+ variants showed a much lower pH optimum compared to PNGase A variants with an optimum pH of 2.6 (for PNGase H+ variant Dj) compared to a pH optimum of 5.0 for PNGase A (from *Prunus dulcis*) (Altmann et al., 1995). Our results here showed that this acidic enzyme is ideal for *in-situ* plant *N*-glycan releasing, and it is compatible with downstream MALDI-MS analysis and detection, as we illustrated by spatially annotating 34 plant characteristic *N*-glycans in soybean root nodules.

The imaging modality of our approach suggests cell-dependent glycosylation of the proteins in the soybean root nodule and points to our previous hypothesis about spatially heterogeneous biochemical processes inside the infection zone (Velickovic et al., 2018). We showed that soybean nodule cells infected by rhizobia show increased *N*-glycosylation compared to non-infected cells, implying that interaction with microbe alters the *N*-linked glycoprotein profile of plant cells. This is the first demonstration of such dependence at the cellular scale and illustrates a novel role of protein glycosylation in host-microbe interaction (Lin et al., 2020). Abundance and localization differences of specific *N*-glycan compositions between WT and *nifH-* mutant soybean nodules highlight their plausible involvement in BNF. One possible explanation for increased *N*-glycosylation in *nifH*- nodules could be the activation of senescence processes that lead to aberrant glycosylation (Cindric et al., 2021). Interestingly, oxidative stress markers including the glycosylated peroxidases were linked to senescence (Evans et al., 1999), but this potential correlation needs to be further validated by other means. Our results indicate that the last stage of complex *N*-glycans synthesis in the trans-Golgi (Strasser, 2016), which leads to Lewis-a glycans, is intensified in the soybean nodules infected with rhizobia without nitrogen-fixing capabilities. Lewis-a containing complex *N*-glycans are evolutionary conserved and have been identified in all plant species (Beihammer et al., 2021). However, despite their ubiquitous occurrence, the biological function of these complex *N*-glycan motifs is currently unknown, which is, in the first place, because the knowledge about proteins bearing Lewis-a is rather scarce. For example, there is strong evidence that Lewis-a glycosylation occurs on enzymes involved in cell wall biosynthesis (Beihammer et al., 2021). A recent review has summarized the contribution of proteomics measurements to our understanding of symbiosis at the molecular level (Larrainzar and Wienkoop, 2017). Our proteomics data also identified the commonly observed stress-related responses in nodulation and provided unique insights into the altered pathways in the WT compared to the *nifH-* mutant that only differ in the nitrogen-fixing capability. Currently, the global proteomics data did not yield sufficient coverage of glycopeptides for us to further investigate the quantitative changes of each type of glycans on a given glycoprotein. We will pursue glycopeptide enrichment in the future, and herein we only attempted to qualitatively correlate the *N*-glycans in MALDI-MSI data to plausible originating glycoproteins by the shared identity of glycans in the proteomics data.

Our proteomic results directly point to nine glycoproteins as potentially major carriers of Lewis-a glycans as possible candidates for further characterization. Glycoproteins with those specific glycans may be involved in mediating efficient BNF or downstream effects during bacteroid infection. Indeed, all these nine proteins have known or predicted functions related to BNF or root development, and it is plausible that their Lewis-a-type glycosylation may be involved in their function. For instance, phytocyanin (I1LWP0), found in both WT and *nifH-* mutant nodules, belongs to a class of plant-specific blue copper proteins that plays a critical role in plant development, including nodule formation. It has been found that exclusion of these genes results in a reduced number of infected cells (Sun et al., 2019). Peroxidase (I1MP39) plays a crucial role in maintaining root nodule redox status, as a basal level of reactive oxygen species (ROS) is essential for initiating nodule developmental processes and maintaining nodule functioning—probably by peroxide action on cell wall proteins (Keyster et al., 2011). Nitrogen-fixing organisms are extremely vulnerable to O_2_ toxicity due to the nitrogenase complex requiring anaerobic conditions for BNF. In synergy with peroxidase and O_2_-buffering systems such as leghemoglobin, uncharacterized glycoprotein oxidoreductase (I1MUX) may serve to maintain proper oxygen levels for BNF (Dalton et al., 1986). Both peroxidase and oxidoreductase are significantly more expressed in the *nifH-*mutant nodules highlighting that the two types of nodules may operate at different O_2_ levels. On the other hand, peroxidases in *Arabidopsis* have also been reported to be involved in lignin biosynthesis, cell wall, and phenylpropanoid metabolism (Herrero et al., 2013; Fernandez-Perez et al., 2015). Phenylpropanoid compounds (e.g., flavonoids, lignin) are known to be related to plant stress response and regulated by glycosylation (Le Roy et al., 2016). As shown in Figure 7, the peroxidase and other interacting proteins in the phenylpropanoid biosynthesis pathway were more abundant in the mutant. The exact role of protein glycosylation in this context is unknown but could be an interesting target for further investigation. Although not directly involved in root maintenance, increased levels of the uncharacterized enzyme with predicted mannosyl-oligosaccharide glucosidase activity (I1K3K7) (Strasser, 2016) could be correlated to the need for a higher glycosylation turnover in mutant root nodules to support elevated glycoprotein synthesis. Uncharacterized protein I1K380, equally distributed in both types of nodules, might be involved in the recognition of peptidoglycans on the rhizobia cell wall or Nod-factor during the initial steps of symbiosis, as this protein contains a highly conserved carbohydrate-binding module, LysM (lysin-like motif) (Madsen et al., 2003). Lastly, germin-like protein (C7S8D5) is also present at the same level in *nifH-* mutant and WT root nodules. These are ubiquitous plant glycoproteins with a conserved β-barrel core that binds manganese, and these proteins are involved in, among other things, proper root development and response to various stress conditions, including microbe infection (Bernier and Berna, 2001). In general, our data indicate that Lewis-a glycan abundance differences observed between plant cells infected with wild-type and mutant rhizobia arise from the proteins involved in phenylpropanoid biosynthesis, *N*-glycan biosynthesis, and glycolysis/gluconeogenesis.

Truncated *N*-glycan structures are also generated in the late stages of the *N*-glycosylation pathway due to the action of β-*N*-acetylhexosaminidases in vacuoles and apoplasts (Liebminger et al., 2011). Based on our MALDI-MSI results, the quantity of these structures is conserved between the two types of nodules. However, insight into the relative abundance of biosynthetic products synthesized in trans-Golgi and vacuole reveals that the truncation yield is higher in the wild type compared to mutant nodules (Figure 3). Unfortunately, we were not able to confidently identify glycoproteins containing truncated *N*-glycans, so the functional role of these glycans is not yet clear in BNF.

Unique distribution patterns of plant *N*-glycans and their significantly increased levels in the soybean nodule variant that cannot efficiently fix nitrogen suggest a functional role of these posttranslational modifications during BNF. However, each glycan detected in our MALDI-MSI data can be released from different proteins in a different cell population. A clear example is illustrated in Figures 4, 5 where morphologically distinct cells have different levels of Hex:5 HexNAc:4 dHex:3 Pent:1. This glycan was found on nine different glycoproteins in the proteomics data (Supplementary Table 2). Although our current glycoproteomic workflow lacks a spatial dimension, it still provides valuable data on the potential connection of the major *N*-glycans to soybean glycoproteins. We showed that the glycans we imaged by MALDI-MS were detected from proteins involved in redox balance crucial for proper nitrogen fixation, root architecture, and plant-microbe recognition.

Dissecting and collecting individual cell types (sclerenchyma, infected cells, non-infected cells, etc.) from the tissue sections and applying our glycoproteomic workflow to the small sample size is the logical next step to elucidate the role of the heterogeneous glycoproteforms during BNF inside infected cells. An alternative approach is developing strategies for *in-situ* glycopeptide enrichment and imaging that will directly link glycans with their protein of origin in the plant cells that share the same glycan composition(s). In addition, rhizobia glycoproteins could be another interesting target to study in the future. Although we expect rhizobia glycopeptides to be present in our proteomics data, they were not investigated at this time due to the lack of comprehensive glycosylation databases. Bacterial *N*-glycan consensus sequences (Nothaft et al., 2009), biosynthesis (Nothaft et al., 2009), and hence enzymatic removal from proteins don't follow the same route as in eukaryotes, therefore posing technical challenges also for MADLI-MSI that are beyond the scope of this study. Whether and how BNF is impaired by remodeling of plant *N*-glycosylation remains to be shown, but our *N*-glycan data can bridge the knowledge gap in the interplay between complex *N*-glycosylation and phytohormones during root landscaping (Frank et al., 2021) that lead to legume nodule organogenesis (Boivin et al., 2016).

## Data Availability Statement

The MALDI imaging datasets for this study can be found in the METASPACE link: https://metaspace2020.eu/project/soybean_nodule_Nglyc_2022. The proteomics data are available at massive.ucsd.edu with MassIVE accession: MSV000088754.

## Author Contributions

DV designed and conducted MALDI-MS imaging experiments and MALDI-MS imaging data analysis and wrote the first draft of the manuscript. MZ supervised the project and designed proteomic experiments. Y-CL performed proteomic experiments and pathway and network analysis. ST performed proteomic data analysis. MV performed sample preparation for MALDI imaging and proteomic analysis. CA and DV initiated *N*-glycan MALDI-MS imaging work on soybean nodules. JV provided PNGase H+. GS provided soybean root nodules. DV and MZ integrated data. All authors revised the manuscript. All authors contributed to the article and approved the submitted version.

## Funding

This research was performed on project awards (10.46936/intm.proj.2020.51671/60000250 and 10.46936/lser.proj.2021.51846/60000343) from the Environmental Molecular Sciences Laboratory, a DOE Office of Science User Facility sponsored by the Biological and Environmental Research program under Contract No. DE-AC05-76RL01830.

## Conflict of Interest

The authors declare that the research was conducted in the absence of any commercial or financial relationships that could be construed as a potential conflict of interest.

## Publisher's Note

All claims expressed in this article are solely those of the authors and do not necessarily represent those of their affiliated organizations, or those of the publisher, the editors and the reviewers. Any product that may be evaluated in this article, or claim that may be made by its manufacturer, is not guaranteed or endorsed by the publisher.
