# Supplementary material for: Spatial Mapping of Plant N-Glycosylation Cellular Heterogeneity Inside Soybean Root Nodules Provided Insights Into Legume-Rhizobia Symbiosis
Source: Front Plant Sci. 2022 May 16;13:869281. doi: 10.3389/fpls.2022.869281 (PMC9150855; doi:10.3389/fpls.2022.869281)
Supplement: Supplementary file 5 [file Data_Sheet_1.DOCX]

Supplementary Material

# Supplementary Figures


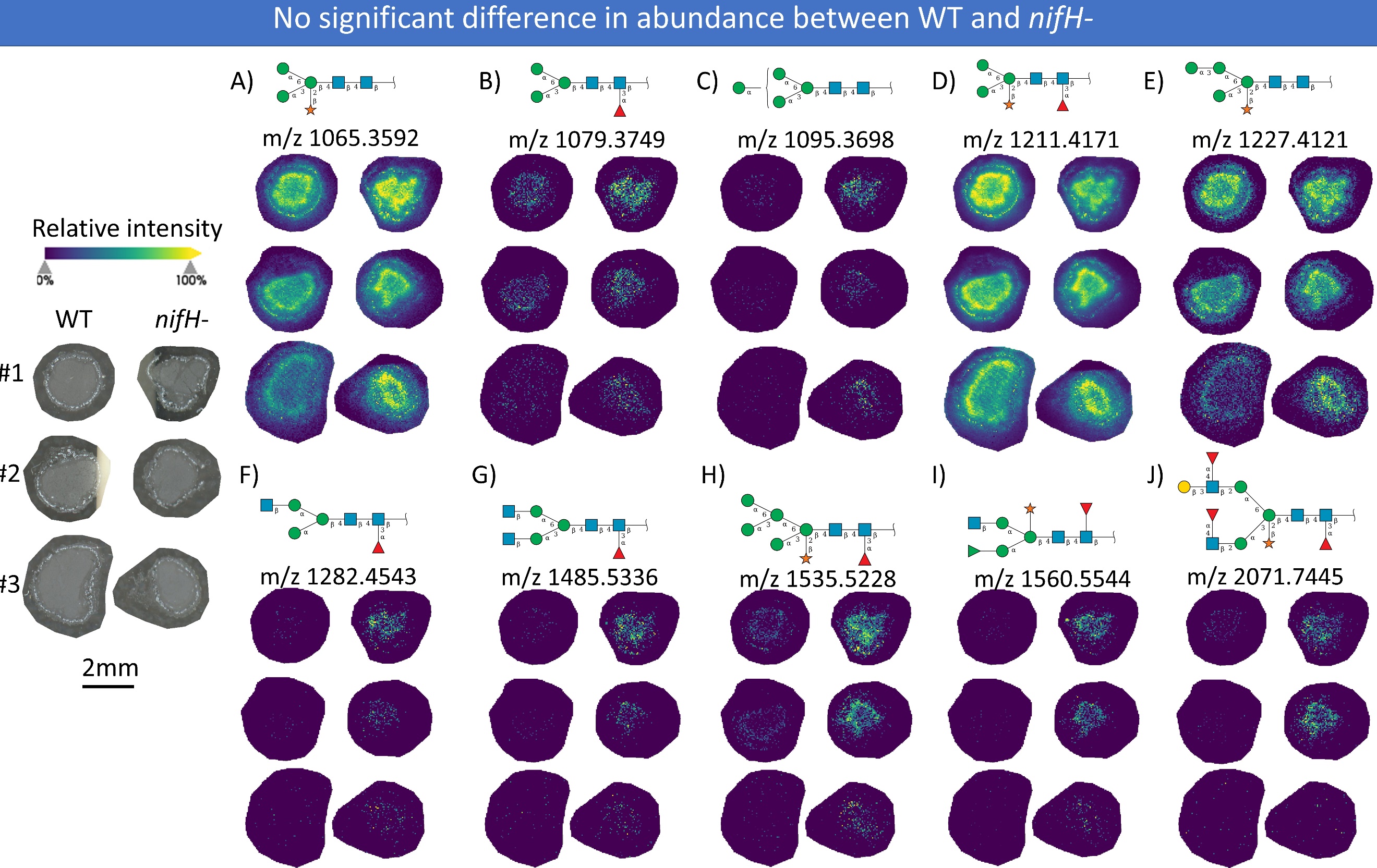


**Supplementary Figure 1.** MALDI MS ion images of *N*-glycans that show no significant difference (with 0.4<AUC<0.6, see Supporting Table 2) in abundance between WT and nifH- nodules over 3 bio-replicates. Tentative structure (SNGF cartoon) for each *N*-glycan composition is depicted above ion images. A) Hex:3 HexNAc:2 Pent:1 B) Hex:3 HexNAc:2 dHex:1 C) Hex:4 HexNAc:2 D) Hex:3 HexNAc:2 dHex:1 Pent:1 E) Hex:4 HexNAc:2 Pent:1 F) Hex:3 HexNAc:3 dHex:1 G) Hex:3 HexNAc:4 dHex:1 H) Hex:5 HexNAc:2 dHex:1 Pent:1 I) Hex:3 HexNAc:3 dHex:2 Pent:1 J) Hex:4 HexNAc:4 dHex:3 Pent:1


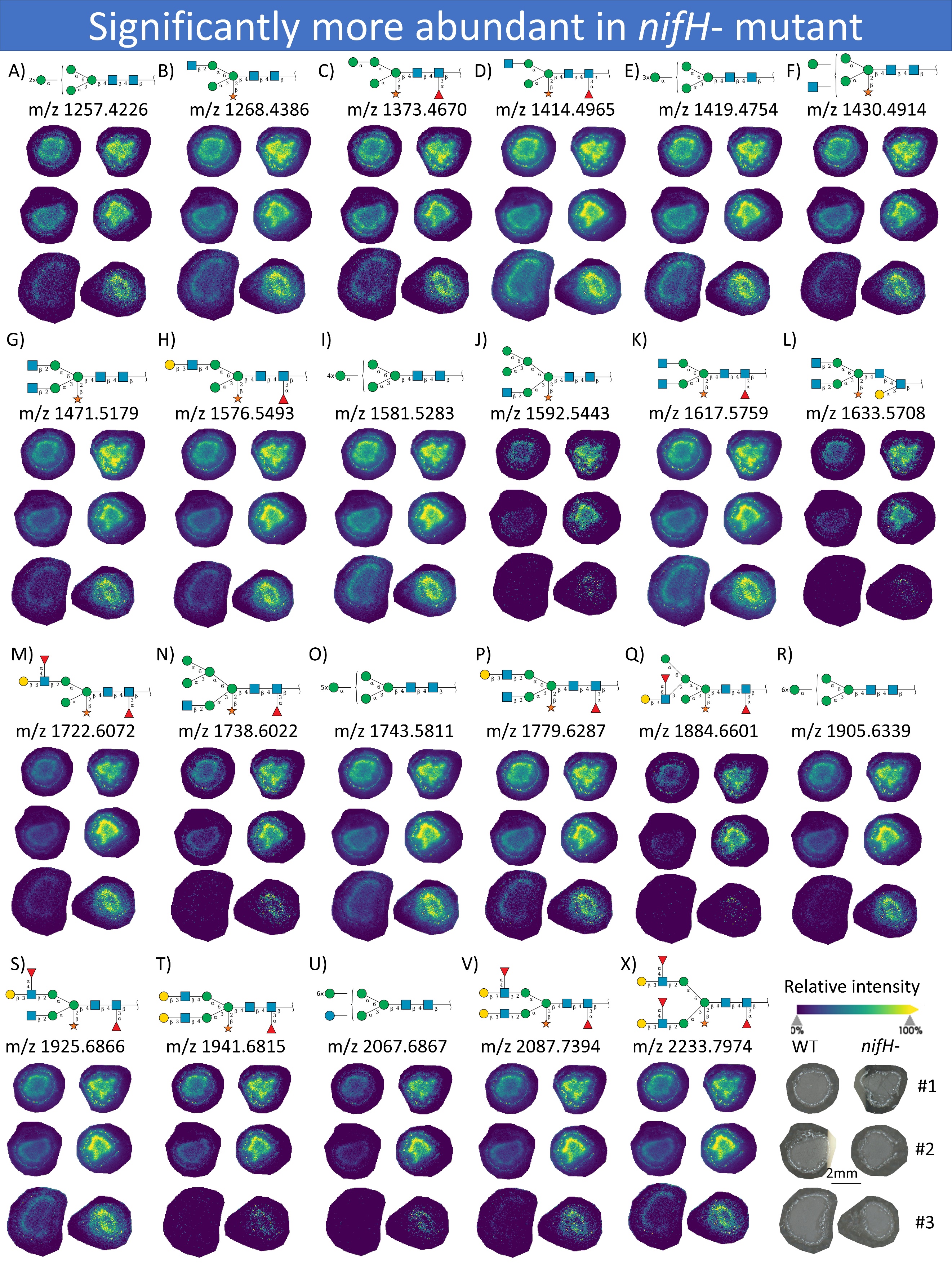


**Supplementary Figure 2.** MALDI MS ion images of *N*-glycans that are significantly more abundant in nifH- than WT nodules (AUC>0.6, See supporting Table) over 3 bio-replicates. Tentative structure (SNGF cartoon) for each *N*-glycan composition is depicted above ion images. A) Hex:5 HexNAc:2 B) Hex:3 HexNAc:3 Pent:1 C) Hex:4 HexNAc:2 dHex:1 Pent:1 D) Hex:3 HexNAc:3 dHex:1 Pent:1 E) Hex:6 HexNAc:2 F) Hex:4 HexNAc:3 Pent:1 G) Hex:3 HexNAc:4 Pent:1 H) Hex:4 HexNAc:3 dHex:1 Pent:1 I) Hex:7 HexNAc:2 J) Hex:5 HexNAc:3 Pent:1 K) Hex:3 HexNAc:4 dHex:1 Pent:1 L) Hex:4 HexNAc:4 Pent:1 M) Hex:4 HexNAc:3 dHex:2 Pent:1 N) Hex:5 HexNAc:3 dHex:1 Pent:1 O) Hex:8 HexNAc:2 P) Hex:4 HexNAc:4 dHex:1 Pent:1 Q) Hex:5 HexNAc:3 dHex:2 Pent:1 R) Hex:9 HexNAc:2 S) Hex:4 HexNAc:4 dHex:2 Pent:1 T) Hex:5 HexNAc:4 dHex:1 Pent:1 U) Hex:10 HexNAc:2 V) Hex:5 HexNAc:4 dHex:2 Pent:1 X) Hex:5 HexNAc:4 dHex:3 Pent:1


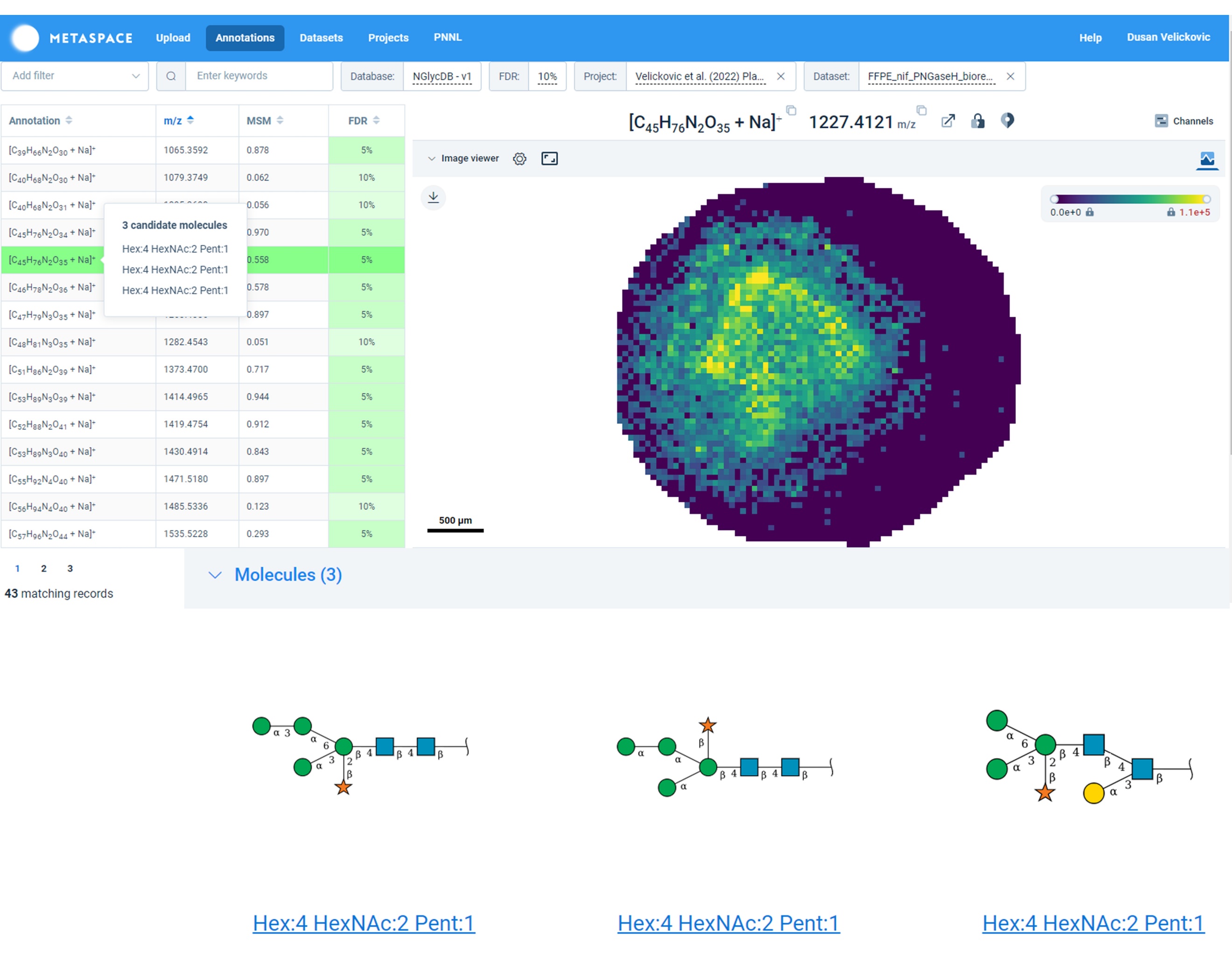


**Supplementary Figure 3.** METASPACE interactive display for browsing isomeric *N*-glycan structures for the given MALDI MS ion image and composition. Structures of isomeric candidates with hyperlinks to GlyConnect can be visualized in the “Molecules” panel.


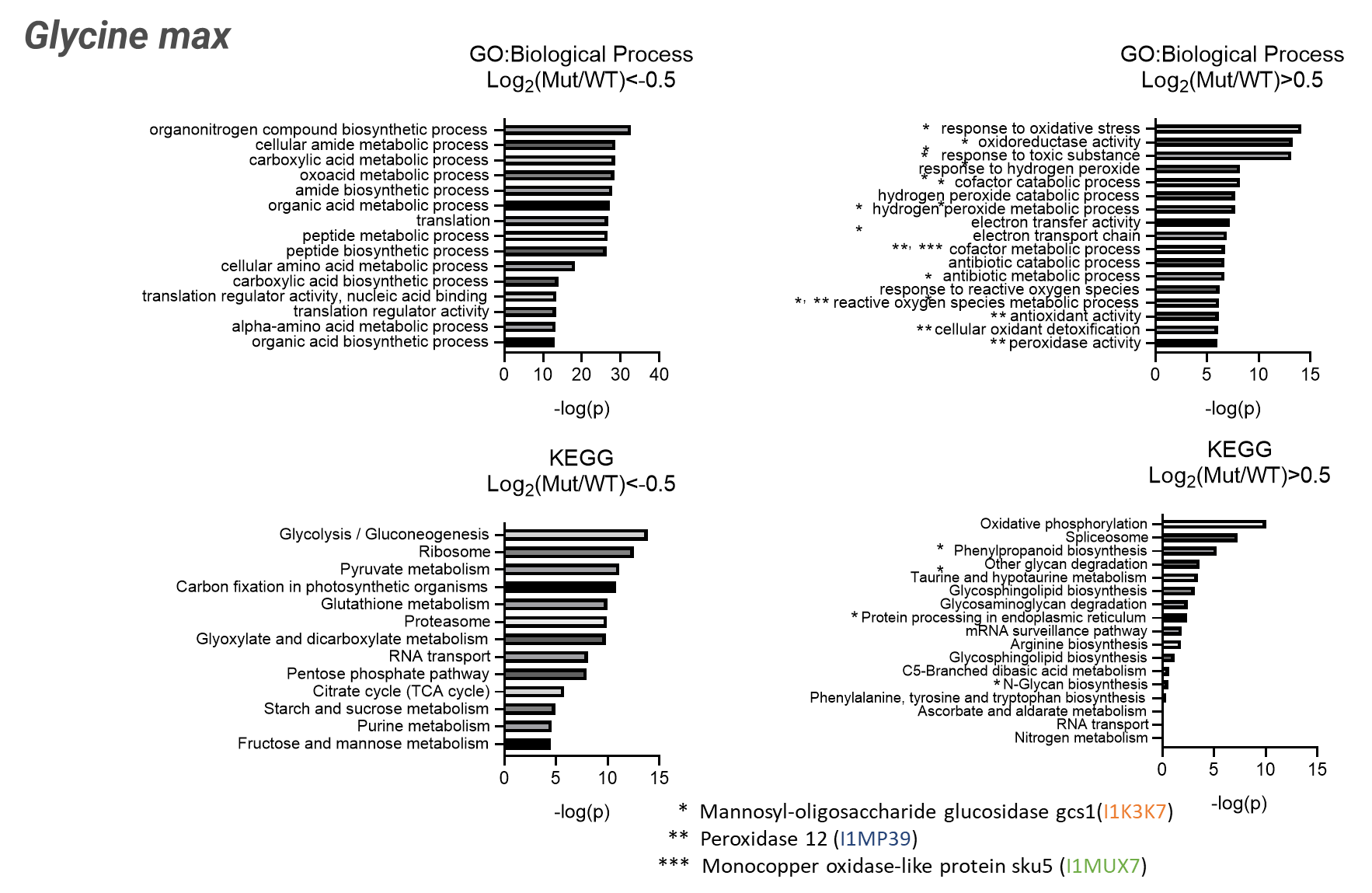


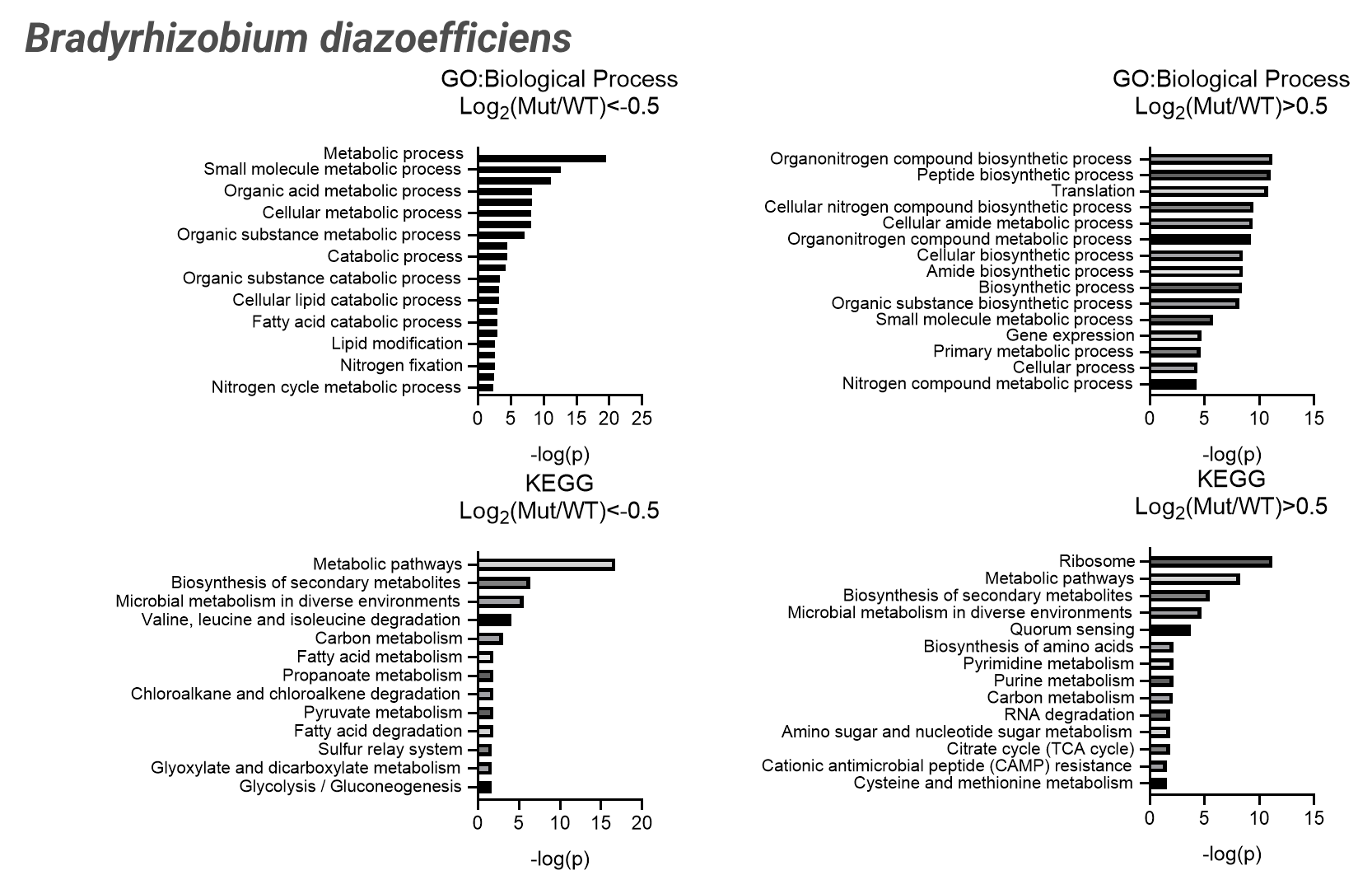


**Supplementary Figure 4.** Pathway chart for proteins overexpressed in the (upper chart) *nifH-* mutant or wild type (WT) rhizobia and (lower chart) soybean infected with wilt type or mutant rhizobia. Detailed list of proteins/genes in each pathway can be found in Supplementary Table 3.

# MS2 spectra of glycopeptides


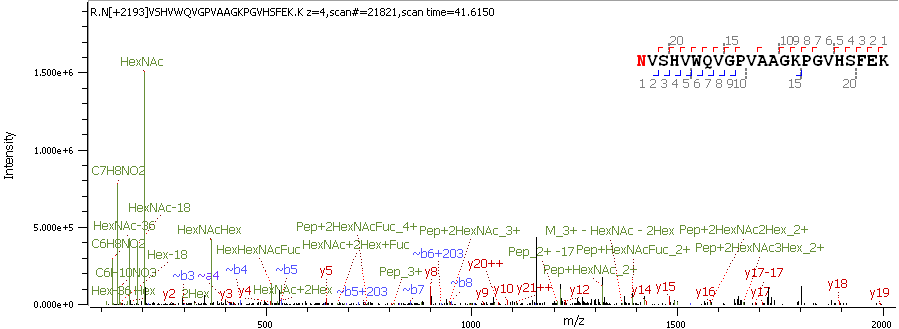


**Supplementary Figure 5a.** MS2 spectrum of glycopeptide identified from C5HU39 containing HexNAc(4)Hex(5)Pent(1)dHex(3) from *nifH-* replicate 3.


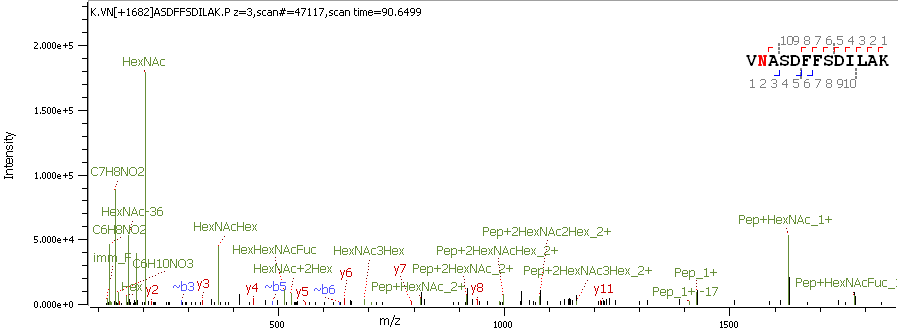

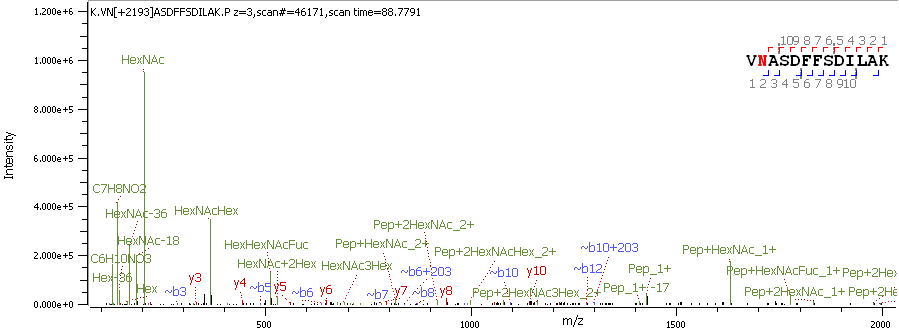


**B**

**A**

**Supplementary Figure 5b.** MS2 spectra of glycopeptides identified from C7S8D5 containing A) HexNAc(3)Hex(4)Fuc(2)Pent(1), or B) HexNAc(4)Hex(5)Pent(1)dHex(3). Spectra obtained from WT replicate 5.


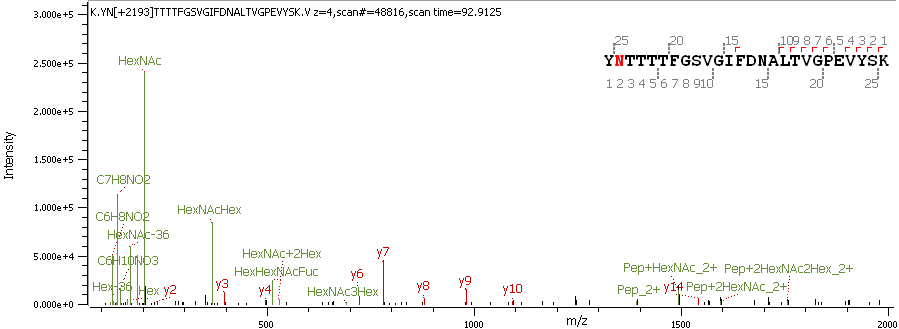


**Supplementary Figure 5c.** MS2 spectrum of glycopeptide identified from I1JL51 containing HexNAc(4)Hex(5)Pent(1)dHex(3) from *nifH-* replicate 5.


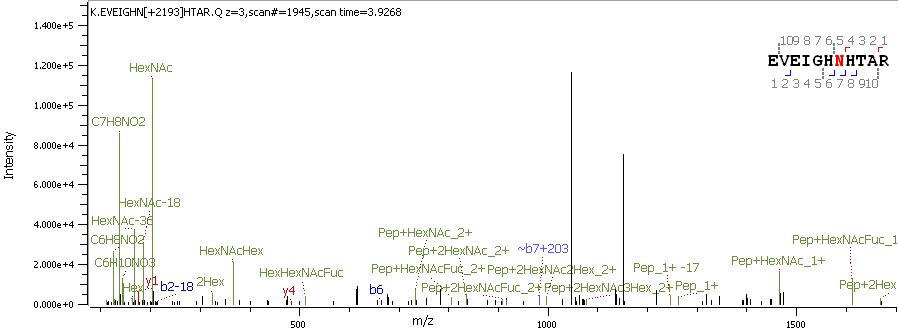


**Supplemental Figure 5d.** MS2 spectrum of glycopeptide identified from I1K3K7 containing HexNAc(4)Hex(5)Pent(1)dHex(3) from *nifH-* replicate 3.


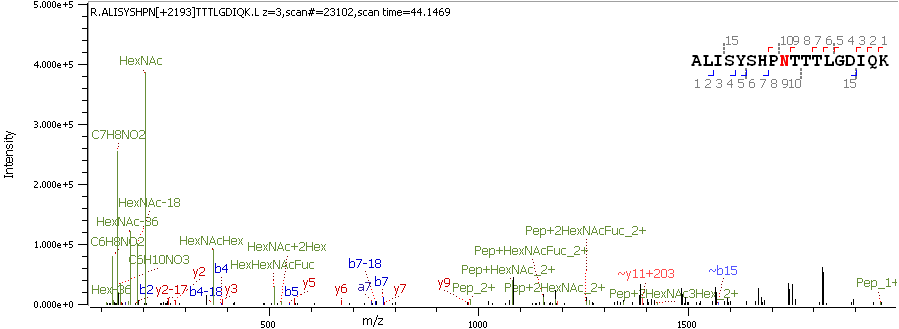

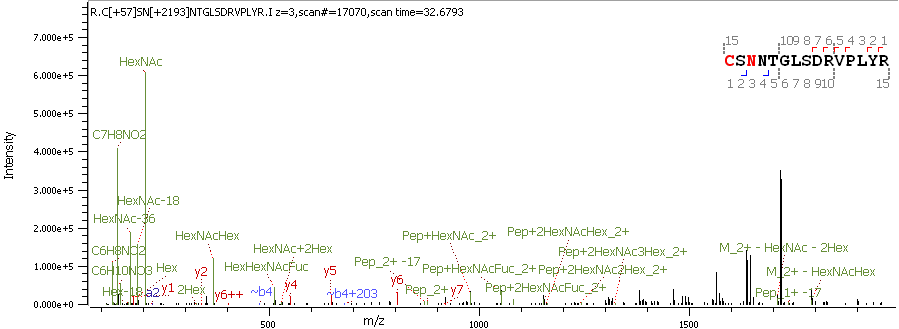

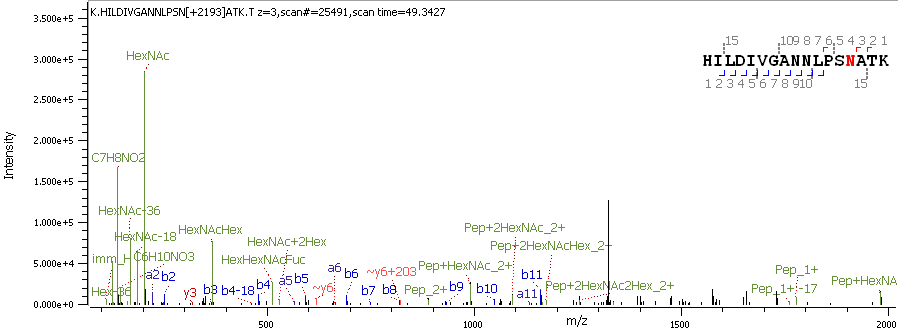


**A**

**C**

**B**

**Supplementary Figure 5e.** MS2 spectra of glycopeptides identified from I1K380 containing HexNAc(4)Hex(5)Pent(1)dHex(3). Spectra obtained from *nifH-* replicate 2 (A and B) and WT replicate 1 (C).


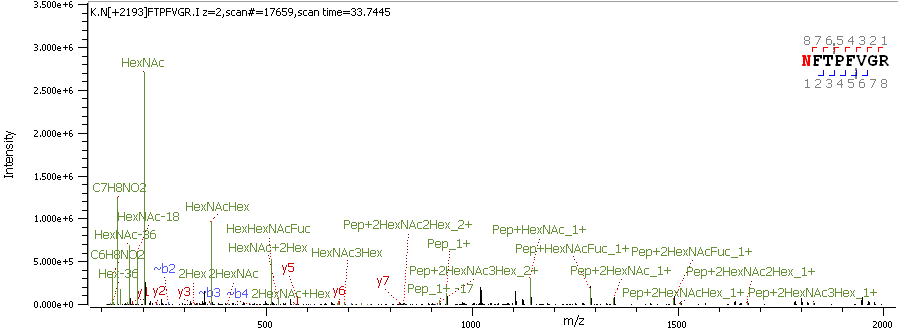


**A**

**B**


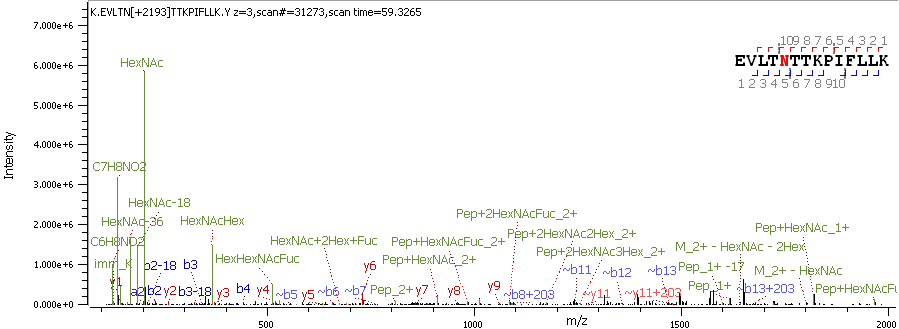


**Supplementary Figure 5f.** MS2 spectra of glycopeptides identified from I1L921 containing A) and B) HexNAc(4)Hex(5)Pent(1)dHex(3) from *nifH-*replicate 3.


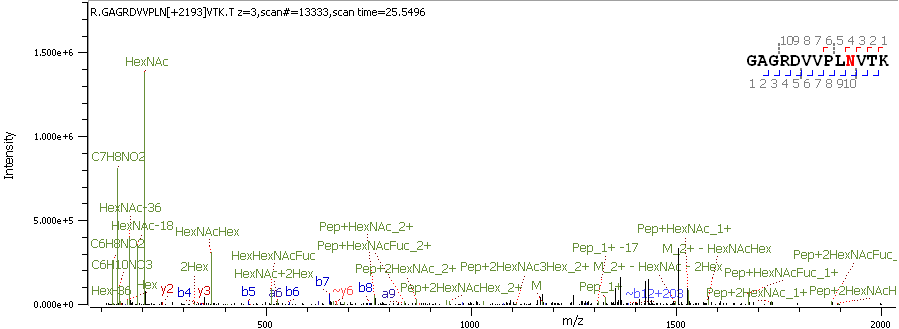

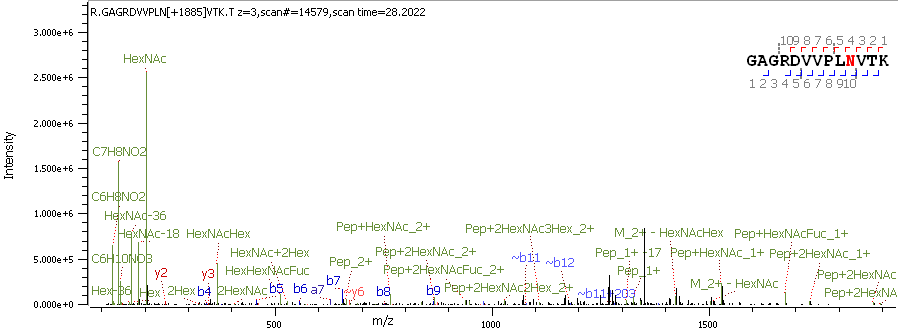


**B**

**A**

**Supplementary Figure 5g.** MS2 spectra of glycopeptides identified from I1LWP0 containing A) HexNAc(4)Hex(5)Pent(1)dHex(3), or B) HexNAc(4)Hex(4)Fuc(2)Pent(1). Spectra obtained from *nifH-*replicate 3 (A) and WT replicate 4 (B).


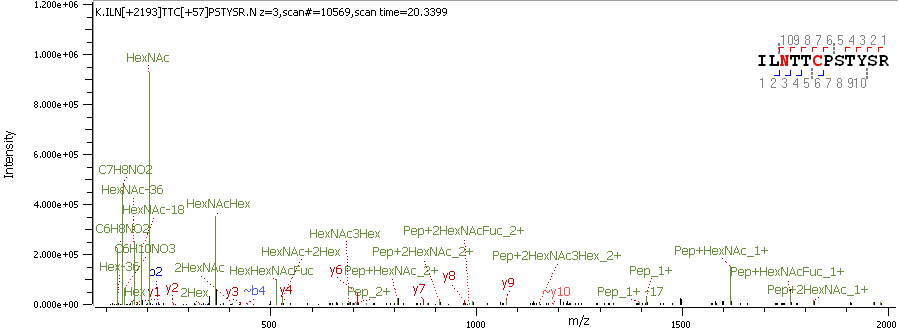


**Supplementary Figure 5h.** MS2 spectrum of glycopeptide identified from I1MP39 containing HexNAc(4)Hex(5)Pent(1)dHex(3) from *nifH-* replicate 4.


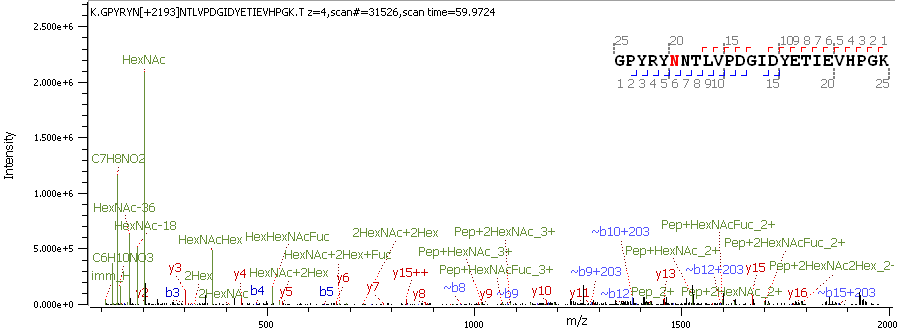


**Supplementary Figure 5i.** MS2 spectrum of glycopeptide identified from I1MUX7 containing HexNAc(4)Hex(5)Pent(1)dHex(3) from *nifH-* replicate 5.


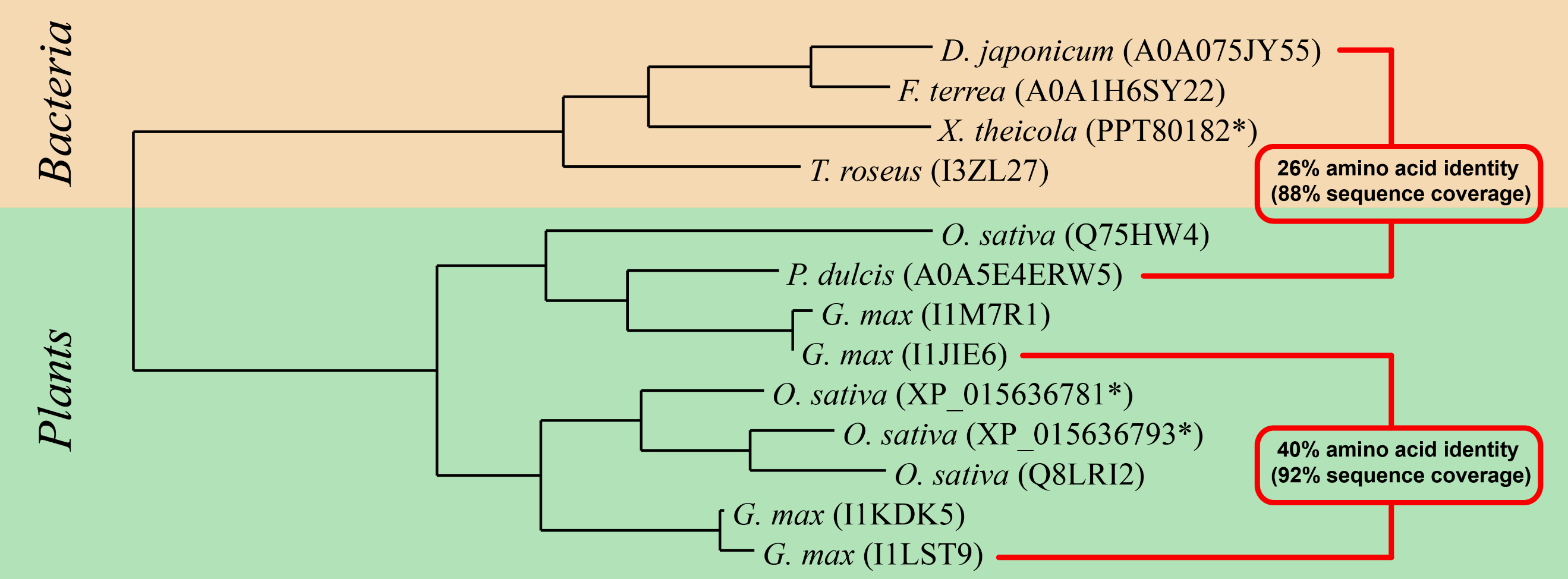


**Supplementary Figure 6**. Phylogenetic relationship between PNGase H+ variants from bacteria and PNGase A variants from almonds (P. dulcis), rice (O. sativa), and soybean (G. max). The PNGase H+ variant from *Dyella japonicum* (Dj, used in this study) and PNGase A from *P. dulcis* (Almond, commonly used to deglycosylate plant glycoproteins) show 26% amino acid identity covering 88% of the protein sequence. In comparison, the two *G. max* PNGase A variants I1JIE6 and I1LST9 show 40% amino acid identity covering 88% of the protein sequence. Uniprot identifiers and Genbank Accession numbers (marked with an asterisk) are shown in brackets. The phylogenetic analysis was performed using “One-Click-Mode” of the Phylogeny.fr online tool. [Ref. DOI: 10.1093/nar/gkn180].
